# DNA Damage Responses in Human Induced Pluripotent Stem Cells and Embryonic Stem Cells

**DOI:** 10.1371/journal.pone.0013410

**Published:** 2010-10-15

**Authors:** Olga Momcilovic, Leah Knobloch, Jamie Fornsaglio, Sandra Varum, Charles Easley, Gerald Schatten

**Affiliations:** 1 Department of Human Genetics, Graduate School of Public Health, University of Pittsburgh, Pittsburgh, Pennsylvania, United States of America; 2 Natural Health Sciences, Seton Hill University, Greensburg, Pennsylvania, United States of America; 3 Pittsburgh Development Center, University of Pittsburgh, Pittsburgh, Pennsylvania, United States of America; 4 Obstetrics, Gynecology & Reproductive Sciences, University of Pittsburgh School of Medicine, Pittsburgh, Pennsylvania, United States of America; Istituto Dermopatico dell'Immacolata, Italy

## Abstract

**Background:**

Induced pluripotent stem (iPS) cells have the capability to undergo self-renewal and differentiation into all somatic cell types. Since they can be produced through somatic cell reprogramming, which uses a defined set of transcription factors, iPS cells represent important sources of patient-specific cells for clinical applications. However, before these cells can be used in therapeutic designs, it is essential to understand their genetic stability.

**Methodology/Principal Findings:**

Here, we describe DNA damage responses in human iPS cells. We observe hypersensitivity to DNA damaging agents resulting in rapid induction of apoptosis after γ-irradiation. Expression of pluripotency factors does not appear to be diminished after irradiation in iPS cells. Following irradiation, iPS cells activate checkpoint signaling, evidenced by phosphorylation of ATM, NBS1, CHEK2, and TP53, localization of ATM to the double strand breaks (DSB), and localization of TP53 to the nucleus of NANOG-positive cells. We demonstrate that iPS cells temporary arrest cell cycle progression in the G_2_ phase of the cell cycle, displaying a lack of the G_1_/S cell cycle arrest similar to human embryonic stem (ES) cells. Furthermore, both cell types remove DSB within six hours of γ-irradiation, form RAD51 foci and exhibit sister chromatid exchanges suggesting homologous recombination repair. Finally, we report elevated expression of genes involved in DNA damage signaling, checkpoint function, and repair of various types of DNA lesions in ES and iPS cells relative to their differentiated counterparts.

**Conclusions/Significance:**

High degrees of similarity in DNA damage responses between ES and iPS cells were found. Even though reprogramming did not alter checkpoint signaling following DNA damage, dramatic changes in cell cycle structure, including a high percentage of cells in the S phase, increased radiosensitivity and loss of DNA damage-induced G_1_/S cell cycle arrest, were observed in stem cells generated by induced pluripotency.

## Introduction

Induced pluripotent stem (iPS) cells are produced by reprogramming somatic cells with a defined set of transcriptional factors. They share numerous characteristics with embryonic stem (ES) cells, such as the ability to undergo self-renewal and differentiation, as well as expression of the same pluripotency markers NANOG, OCT4, SOX2 and SSEA-4 [1]. Therefore, it is possible to envision numerous therapeutic applications for human iPS cells without the ethical challenges involved with human ES cells.

Studies in mouse and human somatic cell reprogramming utilized four transcription factors carried on integrating retroviral vectors. Two cocktails of transcription factors were successfully used: *OCT4, SOX2, KLF4* and *c-MYC* [1], [2], or *OCT4, NANOG, SOX2* and *LIN28* [3]. OCT4, SOX2 and NANOG are master transcriptional regulators of the pluripotent state in embryonic stem (ES) cells [4], [5], [6], [7]. These three transcription factors bind to and activate expression of genes that are involved in maintaining pluripotency, while repressing genes involved in differentiation [8]. OCT4, SOX2, and NANOG also bind to and activate their own genes, creating a positive feedback loop that might “jumpstart” reprogramming [9]. However, *c-MYC, LIN28* and *KLF4* have oncogenic properties and might activate tumor suppression response when expressed in somatic cells. These responses consist of cell cycle arrest, senescence and apoptosis, and may act as a roadblock to reprogramming. Indeed, reprogramming is a very inefficient process with 0.01 – 0.1% success rate [1], [2], [10], suggesting that there are unknown limiting steps necessary for the generation of iPS cells. Low efficiency of somatic cell reprogramming can be partially explained by the activation of TP53 pathway and *INK4/ARF* locus by reprogramming factors. In fact, genetic impairment of *TP53* and *CDKN1A* (p21) levels, as well as *INK4/ARF* locus dramatically increase efficiency of generation of iPS clones, endowing almost every somatic cell with the potential to form an iPS clone [11], [12], [13], [14].

Introduction of transcription factors also increases the γ-H2AX foci [12], which are markers of double strand breaks. Thus, it is possible that TP53 is activated following expression of reprogramming factors by DNA damage, and that oncogenes block reprogramming by activating DNA damage responses.

Since genetic manipulation of *TP53* or *INK4/ARF* locus significantly increases the efficiency of reprogramming, it has been suggested that reprogramming could potentially depend on rare spontaneous mutations or epigenetic silencing of *INK4/ARF* or *TP53* [15]. Another potential explanation is that activation of TP53 by damaged DNA prevents the generation of iPS cells with damaged DNA or DNA repair deficiencies [15]. Taking into account the critical role of TP53 in mediating DNA damage response and tumor suppression, it is critical to investigate the function of TP53 and DNA damage response in iPS cells.

Embryonic stem cells are pluripotent cells that are isolated from the inner cell mass (ICM) of the blastocyst [16]. They represent the *in vitro* counterpart of cells that in developing embryo contribute to embryo proper and some extraembryonic tissues. Similar to cells of the early embryo, ES cells are rapidly dividing. The cell cycle in ES cells is shortened in comparison to somatic cells, mainly due to an abbreviated G_1_ phase and facilitated G_1_ to S transition [17]. Rapid progression through successive rounds of DNA replication and mitotic division may expose ES cells to increased risk of replication errors, which are the most common source of double strand breaks (DSB) in proliferating cells. Double strand breaks can also be induced by various physical (ionizing radiation) and chemical (radiomimetic drugs) agents and represent the most difficult type of DNA damage to repair.

Following introduction of DNA damage, cells elicit a complex DNA damage response comprised of coordinated cell cycle arrest, DNA repair, and in some instances apoptosis. We have previously shown that human ES cells activate ataxia telangiectasia mutated (ATM)-dependent checkpoint signaling cascade, including phosphorylation and nuclear localization of TP53 and arrest in the G_2_/M stage of the cell cycle following irradiation [18]. In this study we extend these findings to iPS cells and focus on understanding the DNA damage response of iPS cells. We investigated activation of checkpoint signaling and induction of cell cycle arrest following exposure of iPS cells to γ-radiation. We further examined double strand break (DSB) repair and contrast the response of iPS cells to ES cells. Finally, we compared the expression of DNA damage signaling and repair gene and protein levels between ES, iPS and differentiated cells. Our results show that reprogramming significantly alters the DNA damage response in iPS cells relative to their parent line, resulting in loss of the G_1_/S checkpoint and a dramatic increase in radiosensitivity. Furthermore, iPS cells share numerous similarities in DNA damage response with ES cells, including G_2_/M cell cycle arrest, efficient DSB repair, and high expression of DNA damage signaling and repair genes.

## Results

### Pluripotency and radiosensitivity in human induced pluripotent stem cells

**Figure 1:**
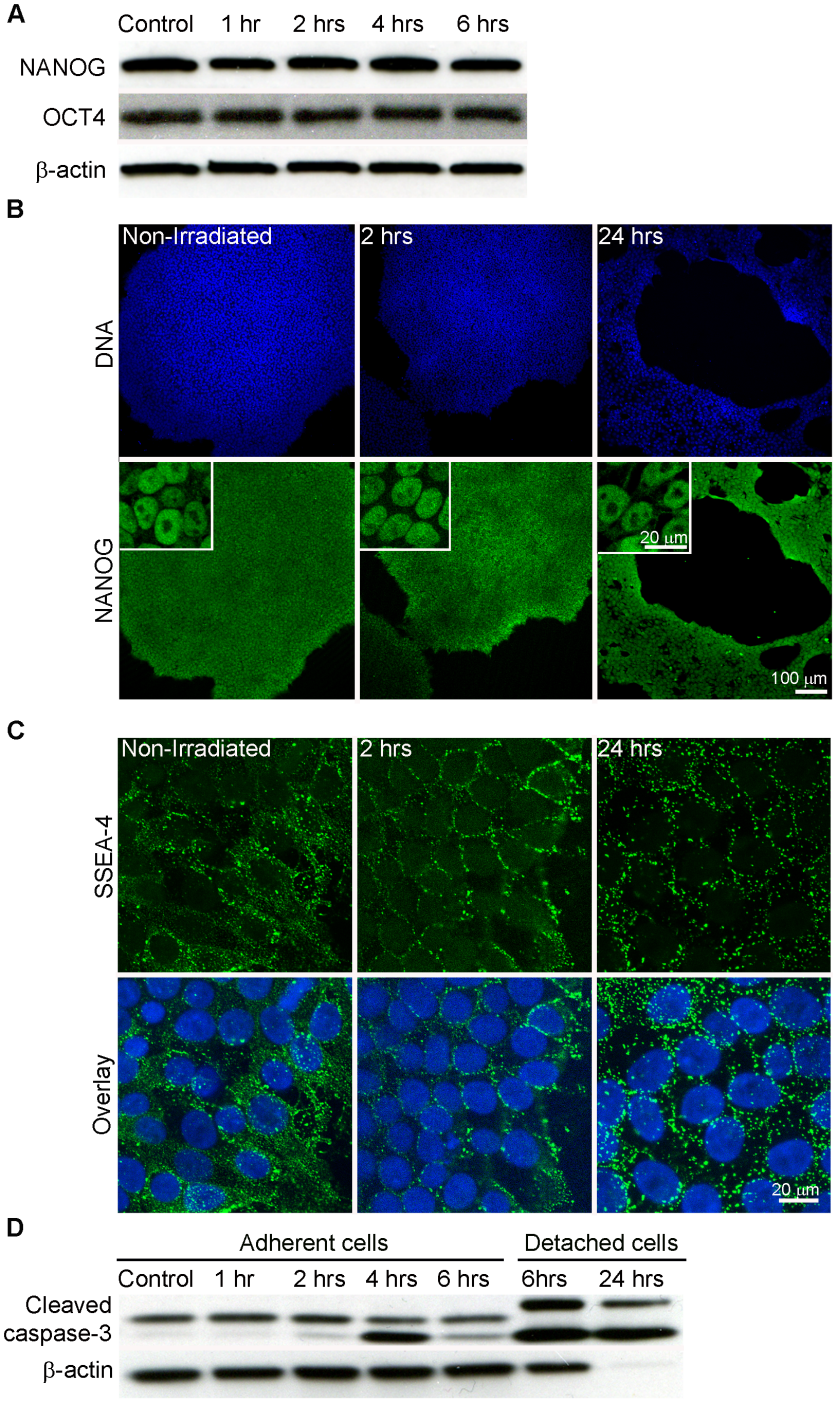
Pluripotency and radiosensitivity of human induced pluripotent stem (iPS) cells. (**A**) Western blot analysis of NANOG and OCT4 demonstrating no change in protein level after irradiation. β-actin served as the loading control. (**B**) Confocal microscopy for NANOG in iPS cells at indicated time periods after irradiation. Inset – zoomed region of a colony displaying nuclear localization of NANOG. Green – NANOG, Blue – DNA, scale bar = 100 µm (20 µm inset). (**C**) Confocal microscopy for SSEA-4 in iPS cells at indicated time periods after irradiation. Green – SSEA-4, Blue – DNA, scale bar = 20 µm. (**D**) Western blot analysis for cleaved caspase-3 after irradiation. Human iPS cells were irradiated, or left untreated, returned to the incubator and recovered for the indicated periods of time. Adherent and detached cells were collected and analyzed separately. β-actin served as the loading control. Note that the level of cleaved caspase-3 increases after irradiation, particularly in detached cells.

Induced pluripotent stem (iPS) cells share numerous similarities with embryonic stem (ES) cells, including self-renewal, differentiation into all three germ layers, and expression of markers found in ES cells, such as OCT4, NANOG, SOX2, SSEA-3 and SSEA-4 [2]. In order to confirm that we are investigating DNA damage response of pluripotent cells we examined expression of the pluripotency markers NANOG, SSEA-4 and OCT4 in both untreated cells and cells irradiated with one Gray (Gy) of γ-irradiation (Figure 1). We decided to use NANOG and SSEA-4 as markers of pluripotency because they were not used in the reprogramming cocktail to derive the AE iPS cell line. Furthermore, in the extremely remote case that irradiation reactivates the reprogramming factors, NANOG and SSEA-4 would still reflect expression of endogenous genes. Both the untreated cell population and those exposed to radiation treatment show expression (Figure 1A) and nuclear localization (Figure 1B) of NANOG, as well as cell surface expression of SSEA-4 (Figure 1C), suggesting that iPS cells retain pluripotency markers after induction of DNA damage. We did not detect a decrease in OCT4 protein levels by Western blot analysis (Figure 1A), confirming these results.

Human and mouse ES cells exhibit profound sensitivity to DNA damaging agents [18], [19]. We also noted substantial detachment of iPS cells from the surface of the cell culture dish by 24 hours following irradiation (Figure 1B). To confirm that cells were undergoing apoptosis, we performed Western blot analysis for cleaved caspase-3, including both adherent and detached cells (Figure 1D). Cleaved caspase-3 began to appear four hours after irradiation in adherent cells and continued to increase for 24 hours, particularly in the detached cells, suggesting that iPS cells undergo apoptosis.

### Activation of checkpoint signaling in irradiated human induced pluripotent stem cells

**Figure 2:**
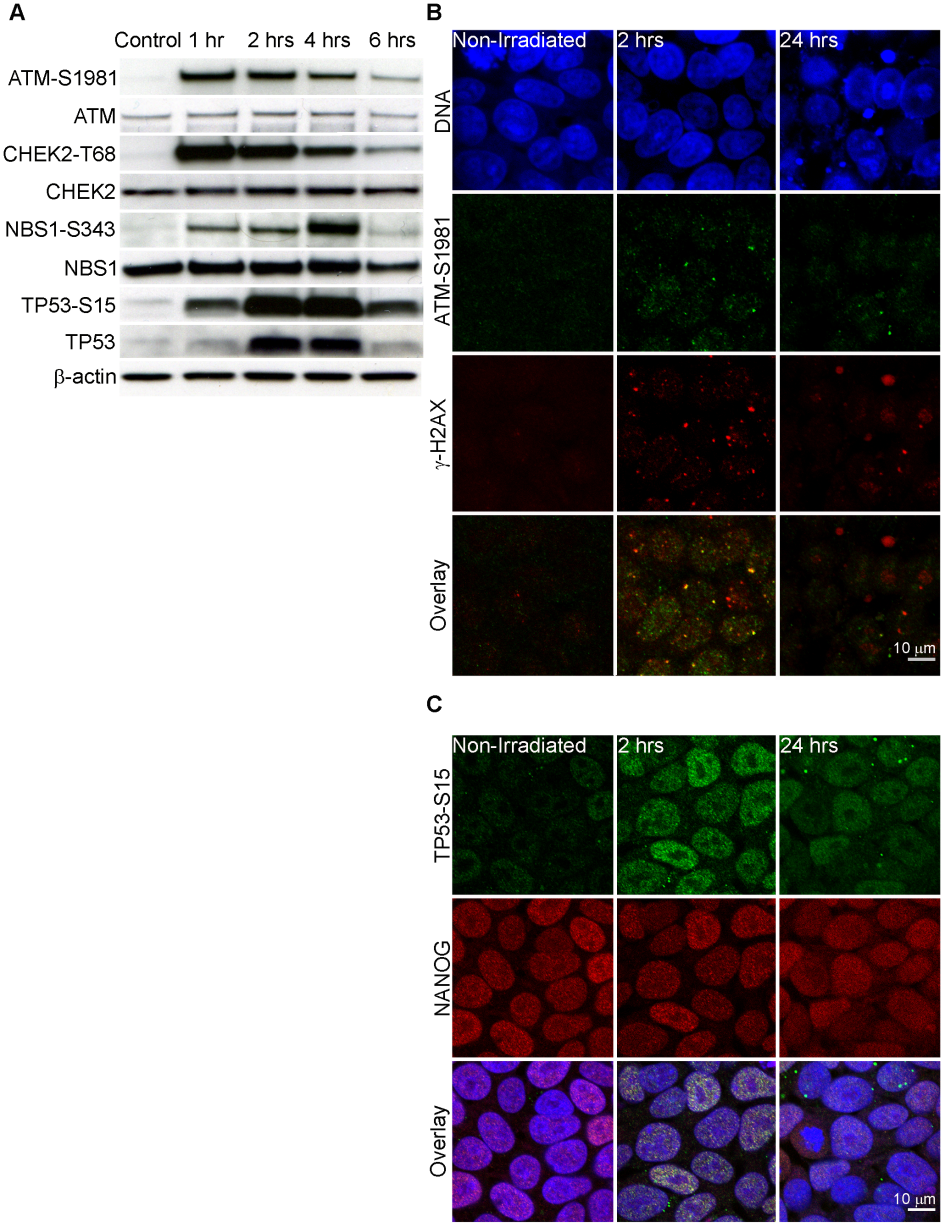
Activation of the checkpoint signaling cascade in human induced pluripotent stem (iPS) cells after irradiation. (**A**) Western blot analysis of ATM-serine 1981, total ATM, CHEK2-threonine 68, total CHEK2, NBS1-serine 343, total NBS1, TP53-serine 15, and total TP53 at indicated time points after γ-irradiation of iPS cells. β-actin served as the loading control. (**B**) Confocal microscopy for ATM and γ-H2AX in iPS cells at indicated time points after irradiation shows co-localization of ATM-S1981 and γ-H2AX after irradiation. Green – ATM-serine 1981, Red – γ-H2AX, Blue – DNA, Yellow – co-localization of ATM-serine 1981 and γ-H2AX. Scale bar = 10 µm. (**C**) Confocal microscopy for NANOG and TP53-serine 15 at indicated time points after irradiation. Red – NANOG, Green – TP53-serine 15, Blue – DNA. Scale bar = 10 µm.

Following introduction of DSB, ATM undergoes auto-phosphorylation at serine 1981 and its kinase function is activated, leading to phosphorylation of numerous downstream targets [20], [21]. We have previously demonstrated that ATM is phosphorylated at serine 1981 and localizes to DSB sites within 15 minutes of γ-irradiation in human ES cells [18]. We tested activation of ATM-dependent checkpoint signaling cascade in two iPS cell lines by Western blot (Figure 2A, Figure S1A) and immunocytochemistry (Figure 2B and 2C) after γ-irradiation. Checkpoint signaling in AE iPS line in response to one Gy of γ-irradiation is depicted in Figure 2, whereas checkpoint signaling following two Gy in IMR-90 iPS line is displayed in Figure S1A (both lines were assayed after both dosages of irradiation, but here we report complementary results). Both dosages induced a strong checkpoint signaling response as evidenced by phosphorylation of ATM and its target proteins. Western blot analysis revealed ATM-serine 1981, CHEK2-threonine 68, NBS1-serine 343, and TP53-serine 15 phosphorylation within one hour of γ-irradiation. ATM-serine 1981 and CHEK2-threonine 68 phosphorylation was highest one hour after irradiation and declined subsequently, staying above steady-state level six hours later. NBS1-serine 343 phosphorylation peaked four hours after irradiation, returning to steady-state levels six hours following irradiation. ATM-dependent phosphorylation of TP53 at serine 15 was highest two hours after irradiation and declined four hours post irradiation. During the same time period the level of total ATM, CHEK2 and NBS1 proteins did not change, whereas the level of total TP53 protein increased following TP53 phosphorylation, suggesting that TP53 is stabilized in response to radiation-induced DNA damage [22], [23]. We also investigated localization of ATM-serine 1981 and TP53-serine 15 in response to radiation exposure. Phosphorylated ATM was localized to sites of DNA damage as detected by co-localization with DSB marker γ-H2AX (Figure 2B). In order to confirm that we were observing the DNA damage response in pluripotent stem cells, we co-stained iPS cells with TP53-serine 15 and NANOG (Figure 2C) and detected nuclear localization of TP53-serine 15 in NANOG-positive cells after irradiation. Additionally, we compared expression of TP53 target genes between non-irradiated and irradiated iPS cells (Figure S2). We detected two-fold or greater upregulation of *CDKN1A (p21), GADD45A, PPM1D, SESN1 (SESTRIN1), SESN2 (SESTRIN2),* and *MDM2* genes, suggesting that TP53 is transcriptionally activated after irradiation. Interestingly, we did not detect upregulation of TP53-dependent apoptosis genes *BAX* and *BCL2*. Collectively, these results are very similar to our previous observations in human ES cells and confirm activation of checkpoint signaling cascade in iPS cells following exposure to γ-irradiation.

### Cell cycle arrest in irradiated human induced pluripotent stem cells

**Figure 3:**
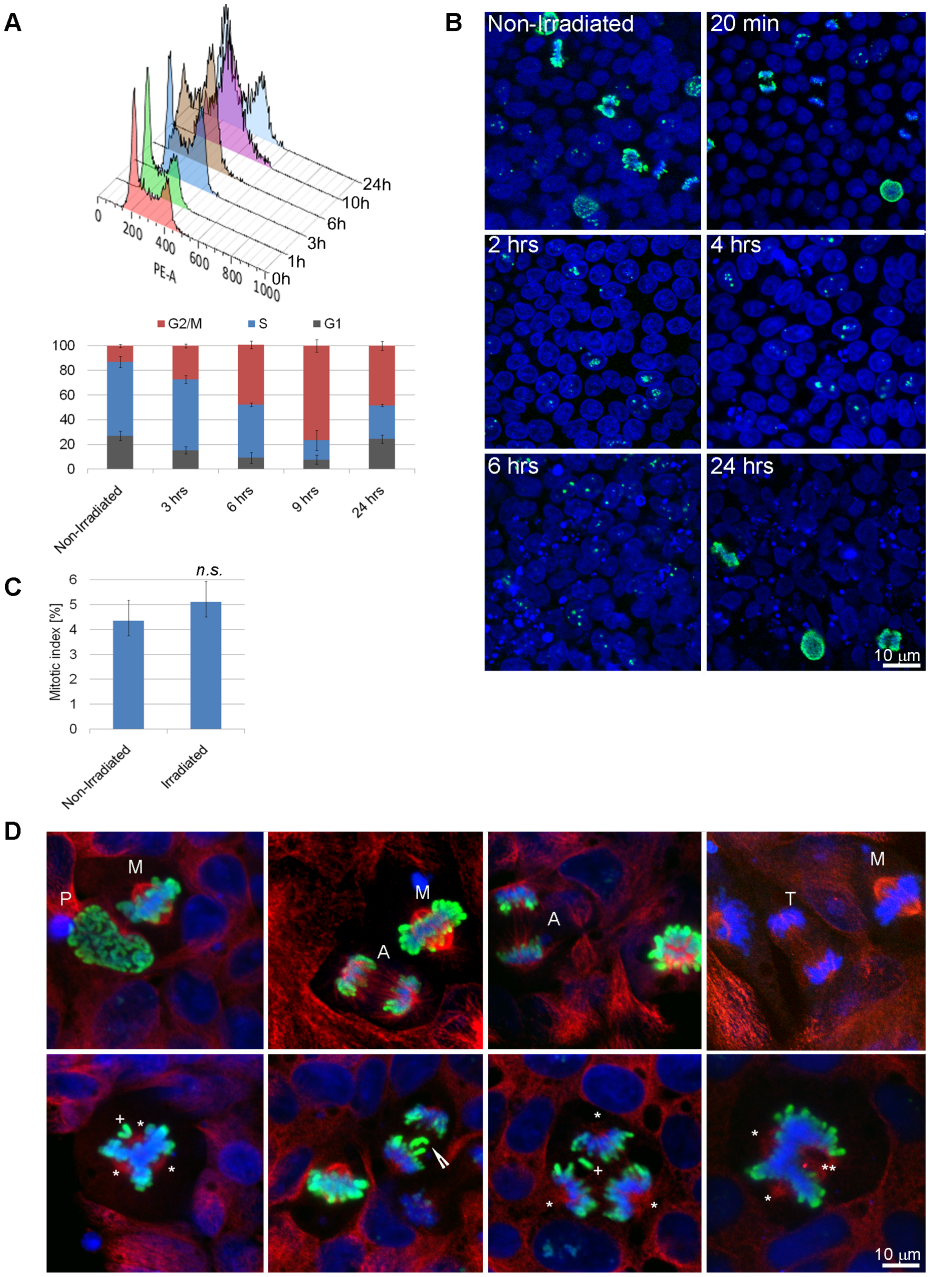
Induction of a temporary G_2_/M cell cycle arrest in irradiated human induced pluripotent stem (iPS) cells. (**A**) Analysis of DNA content of irradiated human iPS cells by flow cytometry using PI. Top panel: DNA histograms displaying cell cycle profiles measured 0–24 hours after one Gy of γ-radiation. Bottom panel: percentages of cells in G_1_, S, and G_2_/M phase of the cell cycle at indicated time points after irradiation. Percentages were calculated using ModFit software (Verity Software House). Results were gated to exclude cellular debris, sub-G_0_ population, and doublets. Data represent the mean ± SEM calculated from three independent experiments. (**B**) Confocal microscopy for phospho-histone H3 after irradiation of human iPS cells. Green – phospho-histone H3, Blue – DNA. Scale bar = 10 µm. (**C**) Quantification of mitotic indexes in non-irradiated cells and 24 hours after irradiation. At least 500 cells per condition were counted in three independent experiments, and statistical difference was determined with χ^2^ test. The data represent mean ± SEM. (**D**) Examples of normal (top row) and aberrant (bottom row) mitotic figures visualized by confocal microscopy after staining for mitotic marker phospho-histone H3. Abbreviations: A – anaphase, M – metaphase, P – prophase, T – telophase. Arrow – lagging chromosome, plus sign – misaligned chromosome, asterisks – pole of mitotic spindle. Green – phospho-histone H3, Red – β-tubulin, Blue – DNA. Scale bar = 10 µm.

We next investigated cell cycle arrest in iPS cells in response to ionizing irradiation by flow cytometric analysis of DNA content using PI (Figure 3A). As expected of self-renewing pluripotent cells, a high percentage of non-irradiated iPS cells were in S phase. After exposure to one Gy of γ-irradiation, iPS cells arrested in the G_2_/M phase of the cell cycle, displaying a similar absence of G_1_/S cell cycle arrest as mouse, non-human primate and human ES cells [18], [24], [25]. Three hours after irradiation a significant drop in the percentage of cells in G_1_ phase was observed, whereas the percentage of cells in G_2_/M increased. This trend continued for six hours after irradiation, and by nine hours after irradiation the majority of cells (77%) were in G_2_/M phase. By 24 hours post-irradiation, cell cycle distribution resembled non-irradiated cells (Figure 3A).

In order to better understand when cells arrest after irradiation (G_2_ or M phase), as well as the events that occur following iPS cells' return to the cell cycle, we performed immunocytochemistry using the mitosis-specific marker histone H3 phosphorylated at serine 10 (phospho-H3). In a population of non-irradiated cells, we observed numerous phospho-H3-positive cells in various stages of mitosis (Figure 3B). Twenty minutes after exposure to one (Figure 3B) or two Gy (Figure S1B) of γ-radiation we still observed mitotic cells, presumably cells that were already undergoing mitosis at the time of irradiation. However, two, four, and six hours after irradiation, we could not detect any mitotic cells, suggesting that iPS cells arrest in the G_2_ phase and do not enter mitosis following irradiation. Mitotic cells start reappearing 24 hours after irradiation (please see Figure 3D for examples), with no statistically significant difference in mitotic index (5.1±0.34%) compared to non-irradiated cells (4.3±0.48%; n = 3, 0.3>*p*>0.2; Figure 3C). Note that cellular debris is present in cell colonies both at 6 and 24 hours after irradiation in accordance with iPS cells' radiosensitivity. In aggregate, both flow cytometry and immunocytochemistry results reveal that iPS cells arrest in G_2_ stage of the cell cycle and resume cell cycle progression by 24 hours following irradiation.

### Double strand break repair in human pluripotent stem cells

The fact that iPS cells resume the cell cycle within 24 hours of DNA damage suggests that DNA breaks are removed by that time and allowing recovery from the cell cycle arrest. We investigated DSB repair by monitoring formation and removal of γ-H2AX foci. Since open chromatin structure can lead to the formation of microscopically visible foci in the absence of exogenous DNA damage[26], and since both human ES and iPS cells have open chromatin structure, we first tested the usefulness of γ-H2AX as a DSB marker in human ES cells by inducing localized damage. We employed a 405 nm laser to induce DNA damage in a specified nuclear region of several ES cells in the colony, as well as in several mouse embryonic fibroblasts (MEF) that were used to co-culture human ES cells (Figure S3). Thirty minutes after laser treatment, we detected γ-H2AX in MEF but only in affected cells and only in the nuclear region that was targeted, confirming our goal behind γ-H2AX testing. In human ES cells, we also detected DNA damage only in cells of colonies that were treated with the laser and not in neighboring cells. However, unlike in MEF cells, γ-H2AX staining was not confined to the affected nuclear region; instead, staining was present in the whole nucleus (Figure S3), perhaps due to highly dynamic chromatin in pluripotent stem cells [27]. Nevertheless, we confirmed that γ-H2AX staining is sensitive enough to detect DNA damage only in laser-treated human ES cells.

**Figure 4:**
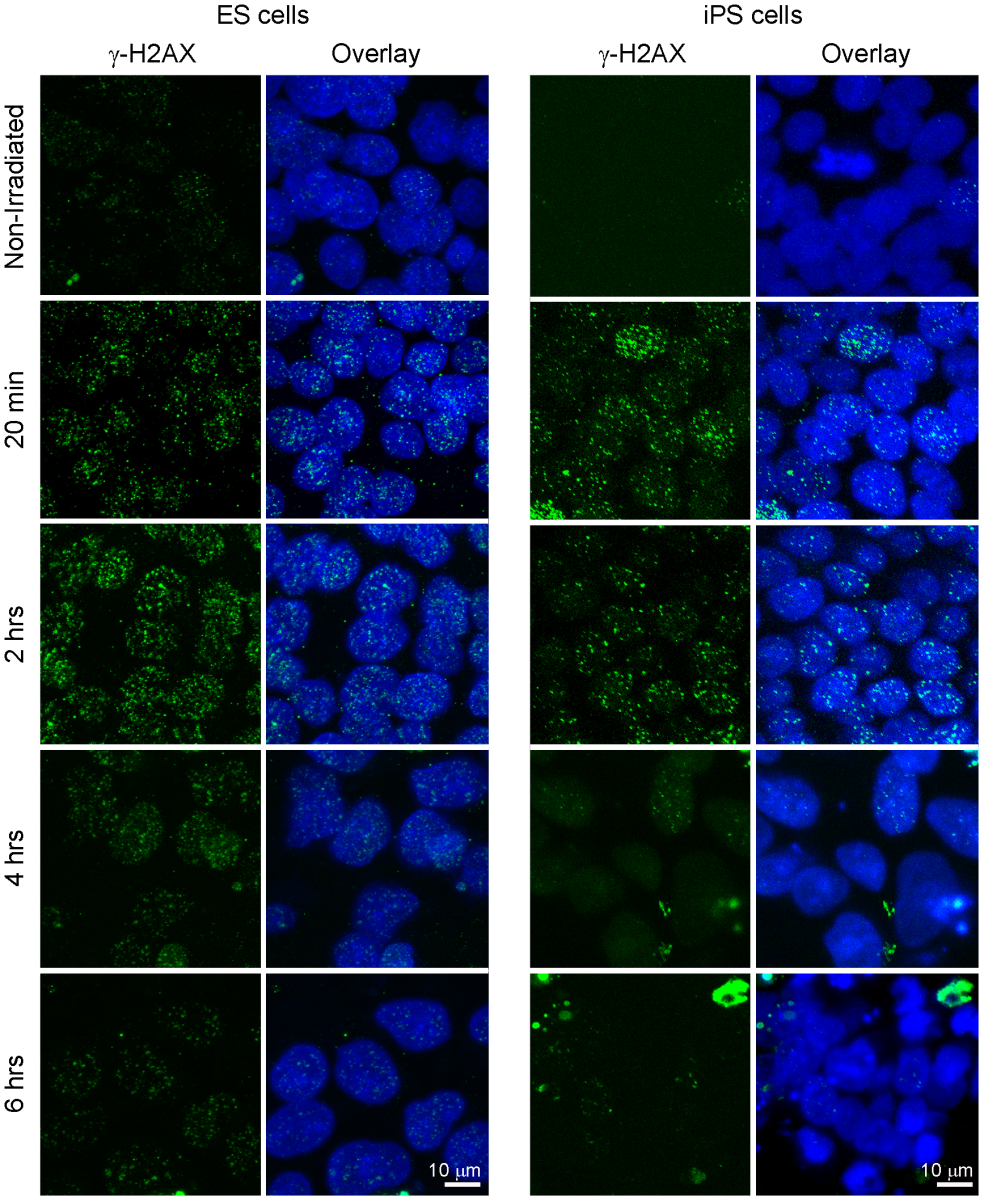
Repair of double strand breaks in irradiated human embryonic stem (ES) and induced pluripotent stem (iPS) cells. Time course immunocytochemistry for marker of double strand breaks γ-H2AX in ES cells (left panel) and iPS cells (right panel). Green – γ-H2AX, Blue – DNA. Scale bar = 10 µm.

We performed a time-course immunocytochemistry for γ-H2AX in irradiated human ES and iPS cells (Figure 4). Non-irradiated human ES and iPS cells were not completely void of γ-H2AX foci, which may reflect endogenous DNA damage. However, within 20 minutes of γ-irradiation we detected strong induction of γ-H2AX foci in both cell types. Over the following four hours, both cell types lost many of these foci, so that by six hours post irradiation the number of γ-H2AX foci had returned to steady-state levels.

**Figure 5:**
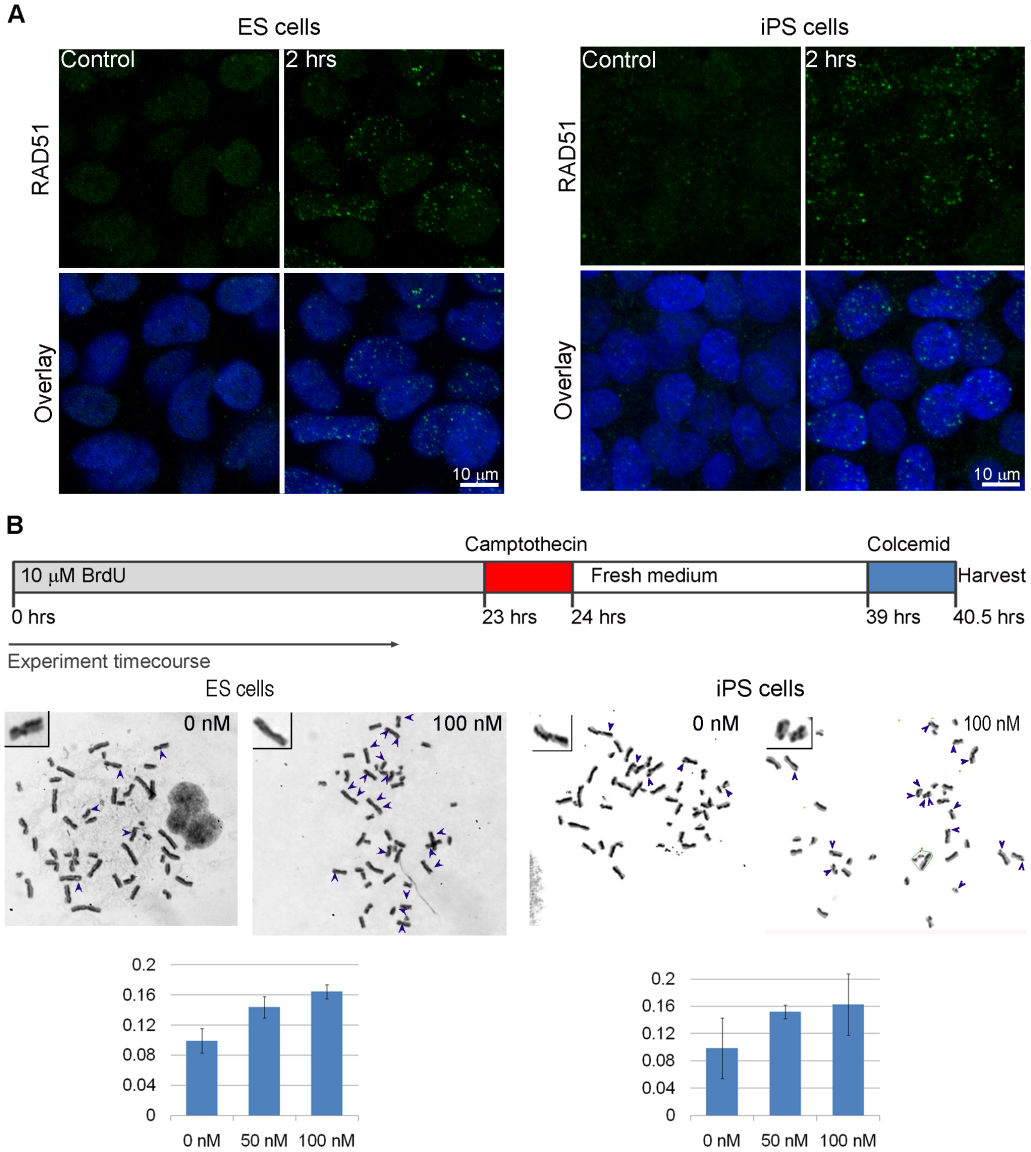
Homologous recombination repair in human stem (ES) and induced pluripotent stem (iPS) cells. (**A**) Confocal microscopy for RAD51 in irradiated human ES cells (left panel) and iPS cells (right panel). Green – RAD51, Blue – DNA. Scale bar = 10 µm. (**B**) Sister chromatid exchanges (SCE) in untreated and camptothecin treated human ES cells (left panel) and iPS cells (right panel). Cells were grown for one cell cycle in the presence of 10 µM BrdU, followed by one hour treatment with 0, 50 or 100 nM camptothecin and 15 hours recovery in the fresh medium. Following 90 minutes of colcemid treatment, cells were harvested for cytogenetic preparation and differential staining with Hoechst 33258 dye and Giemsa was performed. The number of reciprocal SCE was counted in 25 metaphase spreads, and is expressed as number of SCE per chromosome per cell. Error bars represent standard deviation.

Two main pathways for DNA repair include error-prone non-homologous end joining (NHEJ) and error-free homologous recombination repair (HRR). Unlike NHEJ, HRR relies on the presence of the sister chromatid for use as a template for accurate DNA repair and is hence limited to late S and G_2_ phases of the cell cycle. We hypothesized that HRR plays a major role in DSB repair in ES cells due to the fact that, in any given moment, more than 70% of an ES cell population is in the S and G_2_/M phases of the cell cycle [18] when the sister chromatid is available. In order to test this, we exposed ES and iPS cells to γ-radiation and investigated the formation of RAD51 foci (Figure 5A). RAD51 is a recombinase that is essential for HRR and, following induction of DSB, localizes to the foci that are believed to represent sites where HRR takes place. Two hours following irradiation we detected the formation of RAD51 foci in the majority of cells suggesting that both ES and iPS cells repair DSB by HRR. Even more direct evidence of HRR are the sister chromatid exchanges (SCE) that represent a subset of HRR events. We treated ES and iPS cells with 50 and 100 nM camptothecin for one hour to induce breaks in S-phase cells. Camptothecin is a topoisomerase I inhibitor that stabilizes the topoisomerase I – DNA complex. Topoisomerase I-introduced single strand nicks in DNA can be converted into DSB following collapse of the replication fork with stabilized topoisomerase I – DNA complex. Therefore, camptothecin specifically introduces DSB in replicating cells, eliciting HRR. We observed an increase in the number of SCE following 50 nM and 100 nM treatments of ES and iPS cells (Figure 5B). The increase was similar in both cell types, suggesting that iPS cells have similar HRR capacity as ES cells.

### Gene expression analysis in human pluripotent stem cells and differentiated cells

**Figure 6:**
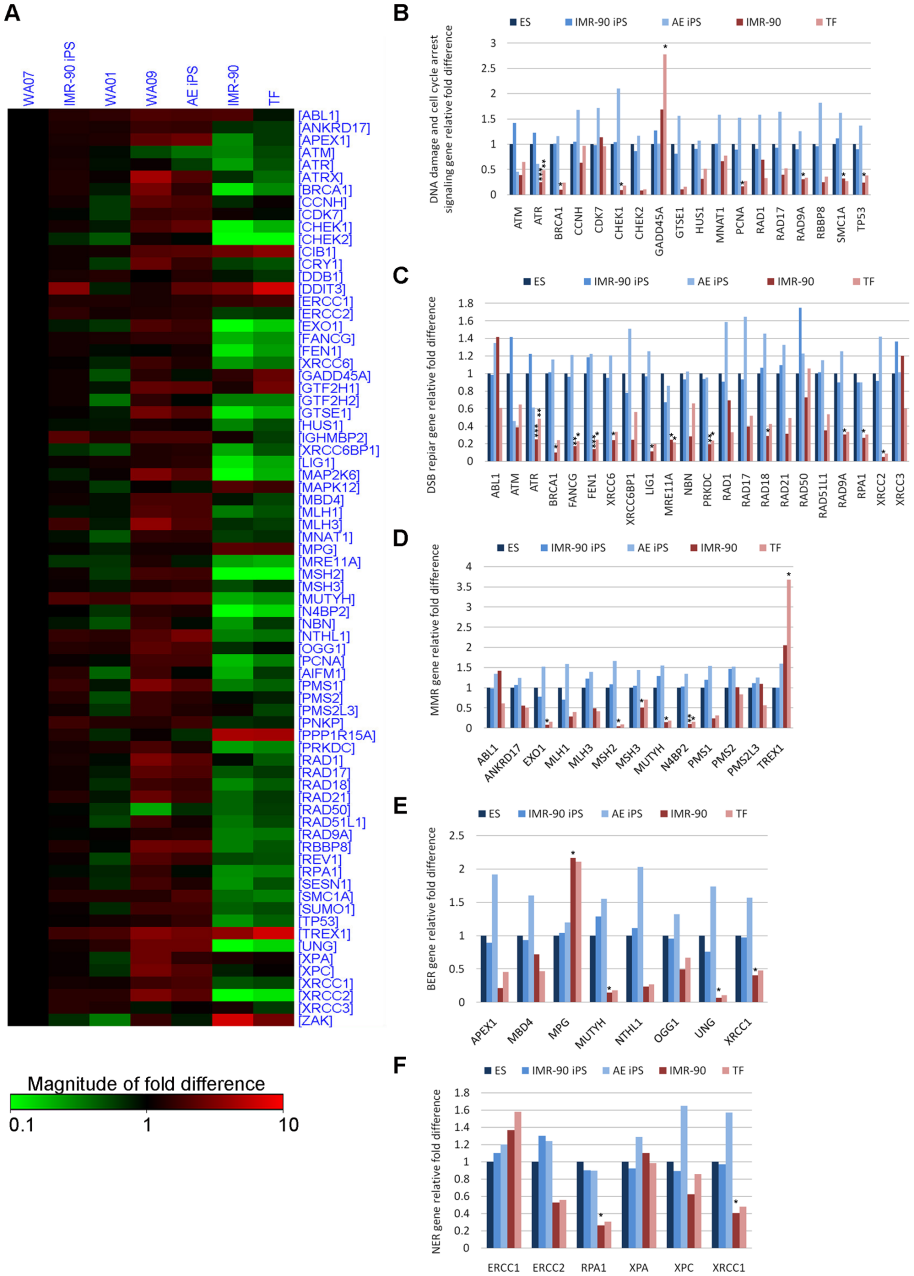
Analysis of DNA damage signaling and repair gene expression in human pluripotent stem cells and differentiated cells. (**A**) Heat map of transcripts analyzed by PCR array. Expression values were normalized over the expression of β-actin and presented as log_10_ of relative changes compared to WA07. In comparison to WA07, genes with higher expression are depicted in red, genes with lower expression are depicted in green, and genes with no difference are depicted in black. The genes were grouped according to the pathway in which they participate: (**B**) DNA damage and cell cycle arrest signaling, (**C**) double strand break (DSB) repair, (**D**) mismatch repair (MMR), (**E**) base excision repair (BER), (**F**) nucleotide excision repair (NER). Expression fold differences were calculated using the -ΔΔCt method relative to averaged ES cell lines and normalized using β-actin as endogenous control. The data presented are from three independent experiments, and asterisks label statistical significance as follows: * 0.01<*p*<0.05, ** 0.001<p<0.01, *** *p*<0.001.

ES cells have been reported to have greater capacity for DNA repair as compared with somatic cells, and have higher expression of some DNA repair genes [28], [29], [30]. We explored this finding in human ES and iPS cells by comparing the expression level of genes participating in DNA damage signaling, cell cycle arrest, and DNA repair across three human ES cell lines (WA01, WA07, WA09), two iPS cell lines (IMR-90 iPS, AE iPS), and two differentiated cell lines (WA07 teratoma fibroblasts [TF] and IMR-90 fibroblasts), using quantitative PCR (qPCR) array (Figure 6). TF and IMR-90 served as differentiated counterparts for ES and iPS cell lines, respectively. Since the ES cell lines did not show any significant difference in gene expression between each other (*p*>0.05, Figure S4), they were grouped together and used as a control for fold difference comparisons. Interestingly, we also could not detect a significant difference in gene expression between ES and iPS lines, although AE iPS line did show increased gene expression relative to ES cells (less than two fold difference, *p*>0.05, Figure S4). The general trend in fibroblasts was reduced gene expression relative to ES and iPS cells (Figure 6A). Out of 57 analyzed genes, 38 (66.7%) showed two-fold or greater downregulation in IMR-90, whereas 31 genes (54.4%) displayed two-fold or greater downregulation in TF. In IMR-90, only two (3.5%) genes were two-fold or more upregulated (*MPG* and *TREX1*), while in TF three genes (5.3%) (*MPG, TREX1* and *GADD45A*) were two or more fold upregulated. Based on the *p*<0.05 cut-off, 23 genes in IMR-90 displayed a statistically significant expression fold difference and nine in TF. These genes also showed at least two-fold difference in comparison to ES lines, so we considered their expression biologically altered. Detailed results of the analysis are available in Table S1.

We grouped genes according to their pathways: DNA damage signaling and cell cycle arrest (Figure 6B), DSB repair (Figure 6C), mismatch repair (MMR; Figure 6D), base excision repair (BER; Figure 6E), and nucleotide excision repair (NER; Figure 6F). The corresponding *p*-values are shown in Table S2. Out of 18 genes involved in cell cycle arrest and DNA damage signaling, seven showed statistically significant lower expression in IMR-90 cells (*ATR, BRCA1, CHEK1, PCNA, RAD9A, SMC1A, TP53*), one showed significant reduction in TF cells (*ATR*), and another showed increased expression in TF (*GADD45A*) (Figure 6B). In a group of 22 DSB repair genes, 13 genes were expressed at significantly lower levels in IMR-90 (*ATR, BRCA1, FANCG, FEN1, XRCC6(Ku70), LIG1, MRE11A, PRKDC(DNA-PKcs), RAD18, RAD9A, RPA1, XRCC2*) and five in TF (*ATR, FANCG, FEN1, MRE11A, PRKDC(DNA-PKcs)*) (Figure 6C). In the group of genes involved in MMR we detected five genes with lower expression in IMR-90 (*EXO1, MSH2, MSH3, MUTYH, N4BP2*) and two in TF (*MSH2, N4BP2*), whereas *TREX1* showed higher expression in TF when compared to ES cells (Figure 6D). Among eight BER genes, three showed reduced expression in IMR-90 (*MUTYH, UNG, XRCC1*), whereas *MPG* showed increased expression in IMR-90 relative to ES cells (Figure 6E). Finally, TF did not show significant difference in expression of genes involved in BER or NER (Figure 6E, 6F). Thus, ES and iPS cell lines display a similar expression pattern for DNA damage signaling and repair genes and exhibit generally elevated expression levels compared to their differentiated counterparts, TF and IMR-90, respectively (Figure 6A). These results suggest that differentiation and reprogramming significantly alter the expression pattern of DNA damage response genes.

**Figure 7:**
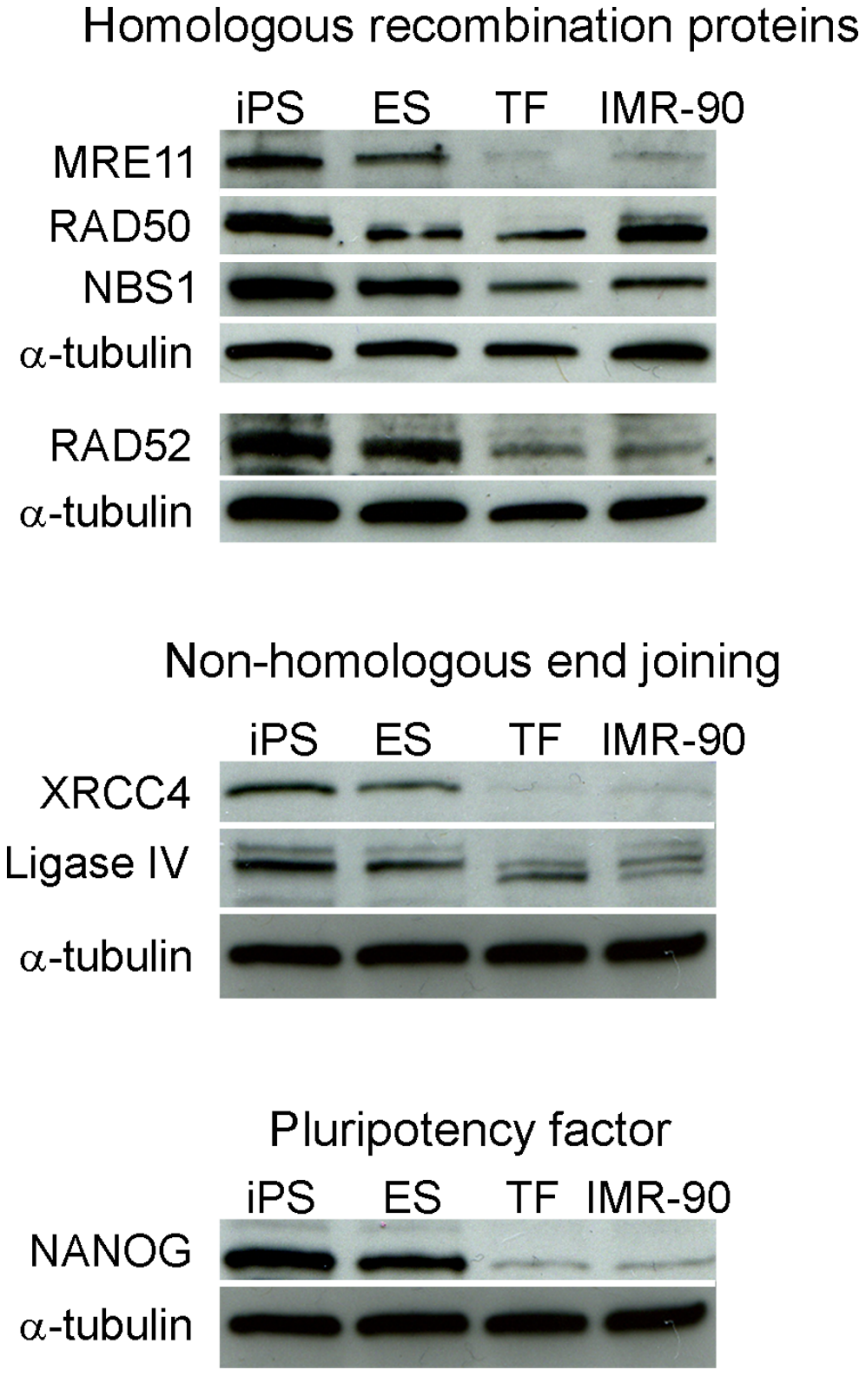
Comparison of DNA repair protein levels between pluripotent stem cells and differentiated cells. Representative Western blot analysis of steady-state level of proteins involved in homologous recombination, non-homologous end joining, and pluripotency maintenance. The Western blots were run in triplicates. α-tubulin served as the loading control.

We also validated gene expression data by comparing DSB repair protein levels between ES, iPS and differentiated cell lines (Figure 7). We detected a lower level of proteins involved in HRR (MRE11, NBS1, and RAD52) and NHEJ (XRCC4 and ligase IV) in TF and IMR-90 cells, compared to ES and iPS cells, thereby confirming gene expression results.

## Discussion

In this study we set forth to investigate the DNA damage response, including checkpoint signaling, cell cycle arrest, apoptosis and DNA repair in iPS cells. We found that the overall response of iPS cells to ionizing radiation is very similar to that of human ES cells. Following activation of the ATM-dependent checkpoint signaling cascade by ionizing radiation, iPS cells arrest cell cycle progression in G_2_ phase and repair DSB. We present evidence for HRR in human ES and iPS cells. Human ES and iPS cells exhibit elevated expression of DNA repair genes, perhaps explaining the high capacity for DNA repair in human ES cells.

Irradiation of iPS cells induced a strong apoptotic response, resulting in cleavage of caspase-3 four hours following radiation treatment and visual loss of iPS cells from the colony 24 hours after irradiation. In our initial experiments we used the same dose of γ-radiation (two Gy) as we did for studies on human ES cells. The iPS cells responded with such a high level of cell death that almost all cells died by 24 hours post-irradiation (data not shown). However, when we performed experiments in human ES cells in parallel with experiments in human iPS cells, we observed similar radiation sensitivity. We attribute this to slightly different culturing method of pluripotent stem cells used in this study (mTeSR™1 medium instead of knock-out medium). We limited the dosage used in this study to one Gy, which elicited a DNA damage response while preserving enough cells to perform experiments. We did not detect such a high level of cell death in IMR-90 fibroblasts, the parent line for IMR-90 iPS cells (data not shown), suggesting that radiosensitivity is specifically associated with pluripotent state. Thus, human iPS cells exhibit profound radiosensitivity, similar to observations in mouse, non-human primate and human ES cells [18], [19], [24], [25].

We also investigated the expression of pluripotency markers NANOG, SSEA-4 and OCT4 in untreated and irradiated iPS cells. As expected, iPS cells show robust expression of NANOG and OCT4 proteins, nuclear localization of NANOG, and cell surface expression of SSEA-4. Similar to previous findings in human ES cells, γ-irradiation does not lead to downregulation of OCT4 and NANOG protein levels during the six hours after irradiation, and confocal microscopy confirmed expression and appropriate localization of NANOG and SSEA-4 up to 24 hours after DNA damage.

We next studied activation of ATM and its target proteins. The kinetics of ATM, CHEK2, NBS1 and TP53 phosphorylation was remarkably similar to observations made in human ES cells. We confirmed canonical localization of ATM-serine 1981 to the sites of DSB by co-localization with γ-H2AX. Double labeling of irradiated iPS cells with NANOG and TP53-serine 15 validated observation regarding activation of checkpoint signaling in pluripotent cells. Finally, examination of expression of TP53 target genes revealed upregulation of *CDKN1A( p21), GADD45A, SESN1, SESN2, MDM2* and *PPM1D*, indicating that TP53 is transcriptionally active. Previous studies showed that *TP53* knockout improves reprogramming efficiency, leading some to question the status of TP53 in iPS cells [15], but our finding suggests that TP53 is active in iPS cells. Therefore, more likely explanation for TP53's role during reprogramming could be prevention of reprogramming of somatic cells with DNA damage.

The analysis of iPS cell cycle distribution revealed a lack of G_1_/S arrest in irradiated iPS cells. Instead, iPS cells arrest in the G_2_ phase of the cell cycle, analogous to data in ES cells. Cell cycle analysis revealed that reprogramming induces changes in the cell cycle, resulting in highly proliferative cell populations mirroring findings in the human ES cells. Since it has been proposed that the G_1_ phase of the cell cycle is a time when ES cells are sensitive to differentiating signals, it is not surprising that reprogramming also leads to loss of G_1_/S cell cycle arrest in iPS cells, which may protect them from differentiation.

Cell cycle analysis showed that cell cycle arrest is temporary and that 24 hours after irradiation iPS cells restart cell cycle progression. This finding suggests that iPS cells repair damaged DNA during this time frame. We followed DSB repair by examining the kinetics of γ-H2AX foci formation and removal. H2AX is a histone variant that is phosphorylated at serine 139 (and referred to as γ-H2AX) by ATM, DNA-PK and ATR at DSB sites and covers megabase-long stretches of DNA surrounding the DSB [31], [32]. In mouse ES cells, γ-H2AX forms visible foci even in the absence of externally-induced DNA damage and, following irradiation, endogenous γ-H2AX foci cannot be clearly distinguished from irradiation-induced foci [26]. This has been attributed to open chromatin structure and global chromatin decondensation that favors formation of visible γ-H2AX foci and limits the use of this marker in following DNA repair in mouse ES cells [26]. Since both human ES and iPS cells also have open chromatin structure, we first validated γ-H2AX as a DSB marker in human ES cells by inducing localized damage with a 405 nm laser. We observed localized γ-H2AX staining in laser-irradiated MEF, but relatively spread out γ-H2AX staining in laser-treated ES cells. This can be explained by dynamic chromatin in pluripotent stem cells [27], so that mobile chromatin distributes damaged DNA from a localized region throughout the nucleus. However, γ-H2AX staining was not observed in untreated MEF and ES cells, justifying the use of γ-H2AX as a DSB marker in human ES cells. When we γ-irradiated ES and iPS cells, we observed γ-H2AX foci forming within 20 minutes of irradiation. The foci disappeared within six hours of irradiation, indicating that DNA repair took place within that time frame, consistent with earlier observations of efficient DNA repair in human ES cells [28].

The two main pathways for repairing DSB are homologous recombination repair (HRR) and non-homologous end joining (NHEJ). Homologous recombination is critically dependent on the presence of the homologous sequence on the sister chromatid, so it typically occurs in the late S and G_2_ phases of the cell cycle. The advantage of HRR is that the original DNA sequence is restored without any loss of information because it uses the sister chromatid as a template, making it an error-free method of repair. In contrast, NHEJ does not require regions of homology and can operate in any stage of the cell cycle. Due to the complex nature of DSB some processing is necessary prior to ligation step and may lead to the loss of genetic information. HRR has long been speculated to be the predominant form of DSB repair in ES cells, and this was recently confirmed in mouse ES cells [29], [33], [34]. Mouse ES cells also demonstrate lower expression of key NHEJ repair factors, such as ligase IV and PRKDC (DNA-PKcs) [29], [34], and show elevated expression or HRR factors (RAD51, Rad54 and RAD52) [29] compared to MEF. Here we show evidence that human ES and iPS cells repair DSB through HRR, by using RAD51 as a surrogate marker for HRR and visualization of SCE.

Gene and protein expression analysis revealed that both human ES and iPS cells have higher expression levels of factors involved in DNA damage signaling and DNA repair than their differentiated counterparts. Maynard and colleagues [28] reported similar data in human ES cells and showed that human ES cells exhibit a higher capacity for repairing multiple forms of DNA lesions. Interestingly, human ES and iPS cells display not only elevated expression of genes and proteins involved in HRR (RAD52, MRE11, NBS1) but also, NHEJ (PRKDC, XRCC6, ligase IV, XRCC4) relative to differentiated cells, unlike in mouse ES cells [29]. Other researchers also described differences in expression levels of NHEJ factors in human ES cells relative to mouse ES cells: human ES cells expressed higher levels of PRKDC and XRCC6 than mouse ES cells and showed proficiency in end-joining, which mouse ES cells lack [34]. It appears that there are species-specific differences between mouse and human ES cells regarding the roles of HRR and NHEJ, that may mirrored by somatic cells: PRKDC activity [35] and XRCC6 expression [36] are higher in human somatic cells relative to mouse cells, and XRCC5 (Ku80) is an essential protein in human but not mouse cells [37]. Nevertheless, the relative contributions and importance of HRR and NHEJ in human ES and iPS cells still need to be clarified.

## Methods

### Ethics statement

All experiments involving animals were approved by the Institutional Animal Care and Use Committees (IACUCs) from the Magee-Womens Research Institute and the University of Pittsburgh, protocol #0803076.

### Cell lines and cell culture

Human ES cell lines WA01, WA07 and WA09 (WiCell Research Institute, Madison, WI), as well as human IMR-90 iPS-clone 1(IMR-90 iPS; WiCell Research Institute) and amniotic epithelium iPS (AE iPS; derived in our laboratory) cell lines were cultured in mTeSR™1 medium (STEMCELL Technologies, Vancouver, BC, Canada) on Matrigel™ (BD Biosciences, Bedford, MA) coated dishes and coverslips. IMR-90 iPS cell line was produced by transducing IMR-90 fibroblasts with retrovirus carrying *OCT4, NANOG, SOX2* and *LIN28* reprogramming factors. AE iPS were derived from amniotic epithelial cells following transduction with retroviral vector carrying all four reprogramming factors *OCT4, SOX2, c-MYC* and *KLF4* that was described previously [38]. Medium was changed daily and human ES and iPS cells were passaged every five to six days using 1 mg/ml dispase (STEMCELL Technologies) following manufacturer's instructions. IMR-90 human diploid fibroblast strain was obtained from American Type Culture Collection (ATCC, Manassas, VA) and cultured in 90% Eagle's Minimum Essential Medium (ATCC) and 10% fetal bovine serum (FBS; Invitrogen, Carlsbad, CA). WA07-derived teratoma fibroblasts (TF) were isolated from a teratoma obtained from mice injected with WA07 cells. Following tumor growth, teratoma was isolated and tissue enzymatically dissociated into single cell suspension. TF were grown in 90% Dulbecco's Modified Eagle Medium (DMEM) supplemented with 10% FBS, 1% nonessential amino acids and 2 mM L-glutamine (all from Invitrogen). Both IMR-90 and TF were enzymatically passaged using TrypLE™ Express (Invitrogen).

### Irradiation

Cells were irradiated with one Gray of γ-irradiation using a Gammacell® 1000 Elite cesium^137^ irradiator (Nordion, Ottawa, Canada). Immediately after irradiation cells were returned to the incubator for recovery until the appropriate time point.

### Immunocytochemistry and Confocal Microscopy

Immunocytochemistry and confocal microscopy were performed as previously described [18]. The following antibodies were used: NANOG (R&D Systems, Inc., Minneapolis), histone-H3-serine 10, TP53-serine 15, SSEA-4 (Cell Signaling Technology, Danvers, MA), ATM-serine 1981 (Epitomics, Burlingame, CA), γ-H2AX (H2AX-serine 139; Millipore, Billerica, MA), β-tubulin (Developmental Studies Hybridoma Bank at the University of Iowa, Iowa City, IA), and RAD51 (EMD4Biosciences, Gibbstown, NJ). Localized DNA damage was induced using a Leica 405 nm laser. The laser beam was targeted at intra-nuclear positions via 63X oil objective lens on Leica TCS-SP2 laser scanning confocal microscope, similar to what has been previously described [39]. A laser intensity of 25% was used. Thirty minutes after damage was induced, cells were fixed and stained with anti-γ-H2AX antibody.

### Western Blot

Human ES and iPS cells were detached from the surface of the cell culture dish by treatment with Accutase™ (Millipore). Cells were washed with PBS and incubated with Accutase™ for two minutes at 37°C. Detached cells were collected, pelleted by centrifugation for five minutes at 200 g, and lysed in RIPA buffer supplemented with 1 mM phenylmethylsulphonyl fluoride (Sigma-Aldrich, St. Louis, MO) and 2x Halt phosphatase inhibitor cocktail (Pierce, Rockford, IL). Fibroblast cell lines were lysed by adding RIPA directly to the flask in which fibroblasts were grown. Western blot analysis was performed as previously described [18], and the following antibodies were used: Ligase IV, RAD50, RAD52, XRCC4 (GeneTex, Irvine, CA), CHEK2-threonine 68, CHEK2, TP53-serine 15, NBS1-serine 343, cleaved caspase-3, β-actin (Cell Signaling Technology), ATM-serine 1981, NBS1 (Epitomics), OCT4, TP53 (Santa Cruz Biotechnology Inc., Santa Cruz, CA), ATM, α-tubulin (Sigma-Aldrich), MRE11 (Millipore), and NANOG (Kamiya Biomedical Company, Seattle, WA).

### Flow Cytometry

Flow cytometric analysis of cell cycle distribution was performed as previously described [18].

### Sister Chromatid Exchanges

For sister chromatid exchange studies, human ES and iPS cells were grown on Matrigel™ as described. Four days after plating the cells, 10 µM 5-bromo-2′-deoxy-uridine (BrdU; Roche Applied Science, Mannheim, Germany) was added to the medium. Following twenty three hours incubation, BrdU-containing medium was aspirated, cells were washed three times with phosphate-buffered saline (PBS; Invitrogen), and medium containing 0, 50 or 100 nM camptothecin (Sigma-Aldrich) was added. One hour following addition of camptothecin, cells were washed three times with PBS and incubated for 15 hours in fresh medium. In order to arrest mitotic cells, 120 ng/ml colcemid (Invitrogen) was added to the medium for 90 minutes. Following colcemid treatment, cells were harvested with Accutase™ and pelleted by centrifugation. Medium was aspirated and one milliliter of prewarmed 0.75 mM KCl (Sigma-Aldrich) was added dropwise while gently tapping the tube. Additional four milliliters of prewarmed hypotonic KCl solution were added and cells were allowed to swell in water bath at 37°C for 20–30 minutes. Cells were prefixed with ten drops of cold fixative (one part acetic acid and three parts methanol; all Sigma-Aldrich) and incubated for five minutes at room temperature. Following centrifugation, supernatant was discarded and fresh cold fixative was added for 30 minutes. This step was repeated two more times, for a total of three fixations. Following the last fixation step, cells were dropped onto slides and left to dry and age for at least one day before staining. Aged slides were rehydrated in PBS and stained with 50 µg/ml Hoechst 33258 (Invitrogen) in dark for 15 minutes. Following a wash in PBS, slides were placed in 2X SSC (0.3 M sodium chloride and 30 mM tri-sodium citrate dihydrate, pH 7.0; Invitrogen) on a 55°C hotplate and exposed to UV at a distance of less than 10 cm for 15 minutes. Slides were rinsed in PBS and stained in 4% Giemsa for 10–15 minutes. Metaphase spreads were visualized using light microscopy and the number of chromosomes and sister chromatid exchanges (SCE) counted. Twenty-five metaphase spreads were analyzed per condition. Results are presented as a number of SCE per chromosome per cell.

### RNA isolation and PCR Arrays

Total RNA was isolated with TRIzol® Reagent (Invitrogen) and genomic DNA was eliminated with the DNA-*free*™ kit (Ambion, Austin, TX) as previously described [18]. Complementary DNA was produced using RT^2^ First Strand Kit (SA Biosciences, Frederick, MD). First, an additional genomic DNA elimination reaction was performed, followed by the first strand cDNA synthesis reaction using one microgram of total RNA. Complementary DNA was subsequently used in the DNA Damage Signaling Pathway PCR array (SA Biosciences) according to the manufacturer's directions. The quantitative PCR reaction was carried out using the following program on Applied Biosystems 7900HT system (Applied Biosystems, Foster City, CA): 10 minutes at 95°C to activate HotStart DNA polymerase, followed by 40 cycles at 95°C for 15 seconds and at 60°C for one minute. The PCR array (PAHS-029) contained pre-dispensed primer sets that include 84 DNA damage signaling pathway genes, five housekeeping genes for normalization of PCR array data, one genomic DNA contamination control, three reverse transcription controls, and three PCR positive controls. The TP53 signaling array (PAHS-027) had similar layout, except that it contained 84 genes involved in TP53 signaling pathways. Data were analyzed using SDS 2.2.2 software and the SA Biosciences data analysis tool (http://sabiosciences.com/pcrarraydataanalysis.php). We excluded from the DNA damage signaling array data analysis of ten genes with Ct values above 30 (*BTG2, CIDEA, DMC1, GADD45G, GML, IP6K3, PCBP4, RAD51, SEMA4A, TP73*). Expression fold differences were calculated using the -ΔΔCt method. β-actin was used as an endogenous control to calculate ΔCt values (Ct_gene of interest_ – Ct_β-actin_). Gene expression fold differences were calculated relative to the averaged ΔCt value in embryonic stem (ES) cell lines (WA01, WA07, WA09), as the ratio of 2^-ΔCt^ between the cell line of interest and ES cells (2^-ΔCt cell line of interest^/2^-ΔCt ES cells^).

### Statistical analysis

Means and SEMs were calculated, and the statistical significance for mitotic index before and after irradiation was determined by χ^2^ test. The significance was determined at *p*<0.05. *p*–values for SA Biosciences PCR arrays were calculated using a web-based analysis software available at http://sabiosciences.com/pcrarraydataanalysis.php. The *p*-values were calculated based on a two-tailed Student's *t*-test, and statistical significance was determined at *p*<0.05.

## Supporting Information

Figure S1 Activation of checkpoint signaling and cell cycle arrest in human induced pluripotent stem (iPS) cells. (A) Western blot analysis of ATM-serine 1981, total ATM, CHEK2-threonine 68, total CHEK2, NBS1-serine 343, total NBS1, TP53-serine 15, and total TP53 at indicated time points after two Gy of γ-radiation of iPS cells. β-actin served as the loading control. (B) Confocal microscopy for phospho-histone H3 after irradiation of human iPS cells. Red - phospho-histone H3, Blue - DNA. Scale bar = 10 µm.
(3.02 MB TIF)

Figure S2 Expression of TP53 target genes in irradiated human induced pluripotent stem (iPS) cells. Human iPS cells were irradiated with one Gy and gene expression fold changes were calculated at indicated time points using the -ΔΔCt method relative to non-irradiated iPS cells and normalized using β-actin as endogenous control.
(0.15 MB TIF)

Figure S3 Induction of localized DNA damage in mouse embryonic fibroblasts (MEF) and human embryonic stem cells (hESC). A 405 nm laser was used at 25% intensity to induce DNA damage in defined nuclear region of MEF and hESC. Thirty minutes following DNA damage, cells were fixed and stained for double strand break marker γ-H2AX. Note that only cells affected with laser show γ-H2AX staining. Green - γ-H2AX, Blue - DNA. Scale bar = 10 µm.
(1.26 MB TIF)

Figure S4 Gene expression comparison between pluripotent stem cell lines. Volcano plots represent p-values (Y-axis) for observed difference in gene expression (X-axis). Blue horizontal line represents position of -Log10(0.05) for easier visualization of significant (p<0.05) difference. Horizontal lines represent two fold boundaries: on the left are genes with more than two fold downregulation (green), and on the right are genes with more than two fold upregulation (red) in comparison to the control line. Genes in between vertical lines show a less than two fold difference in gene expression and are depicted in black. Dots represent genes on the array.
(0.32 MB TIF)

Table S1 Detailed analysis of DNA Damage Signaling PCR Array.
(0.83 MB DOC)

Table S2 p-values for fold difference in gene expression in designated cell line relative to human embryonic stem cells.
(0.10 MB DOC)
