# Supplementary figures and images for: DNA Damage Responses in Human Induced Pluripotent Stem Cells and Embryonic Stem Cells
Source: PLoS One. 2010 Oct 15;5(10):e13410. doi: 10.1371/journal.pone.0013410 (PMC2955528; doi:10.1371/journal.pone.0013410)

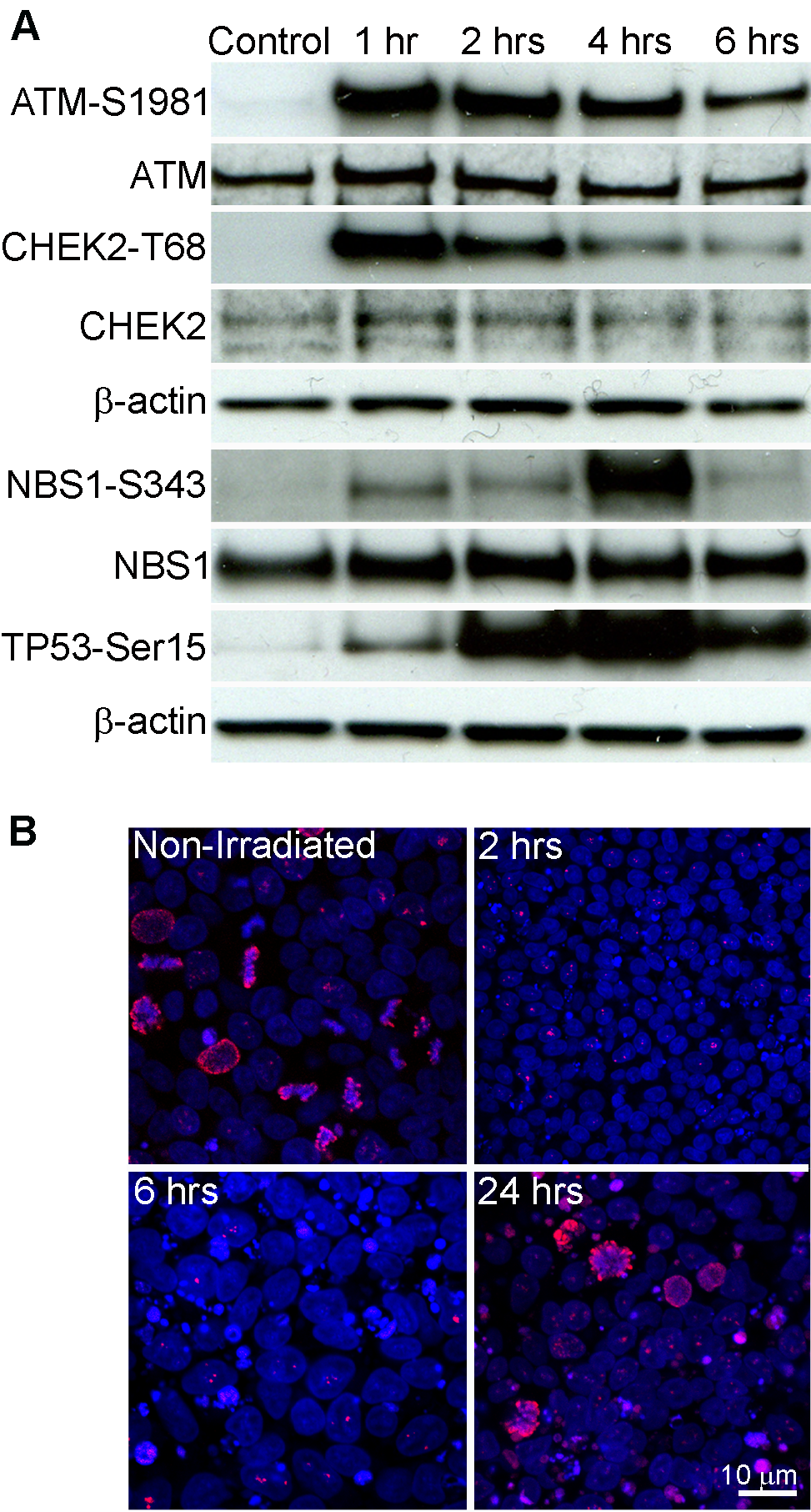

Supplement: Figure S1 — Activation of checkpoint signaling and cell cycle arrest in human induced pluripotent stem (iPS) cells. (A) Western blot analysis of ATM-serine 1981, total ATM, CHEK2-threonine 68, total CHEK2, NBS1-serine 343, total NBS1, TP53-serine 15, and total TP53 at indicated time points after two Gy of γ-radiation of iPS cells. β-actin served as the loading control. (B) Confocal microscopy for phospho-histone H3 after irradiation of human iPS cells. Red - phospho-histone H3, Blue - DNA. Scale bar = 10 µm. (3.02 MB TIF) [file pone.0013410.s001.tif]

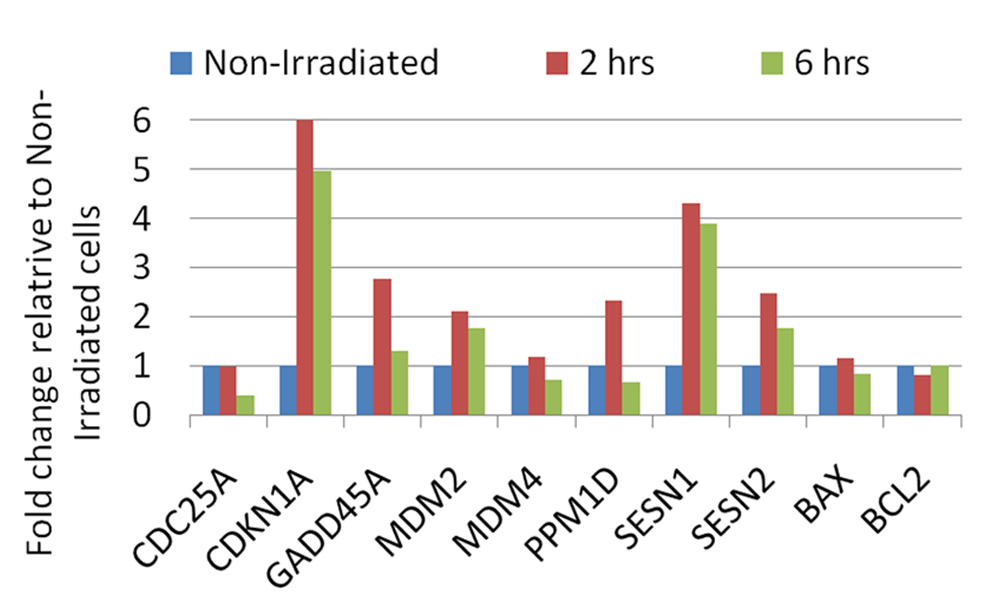

Supplement: Figure S2 — Expression of TP53 target genes in irradiated human induced pluripotent stem (iPS) cells. Human iPS cells were irradiated with one Gy and gene expression fold changes were calculated at indicated time points using the -ΔΔCt method relative to non-irradiated iPS cells and normalized using β-actin as endogenous control. (0.15 MB TIF) [file pone.0013410.s002.tif]

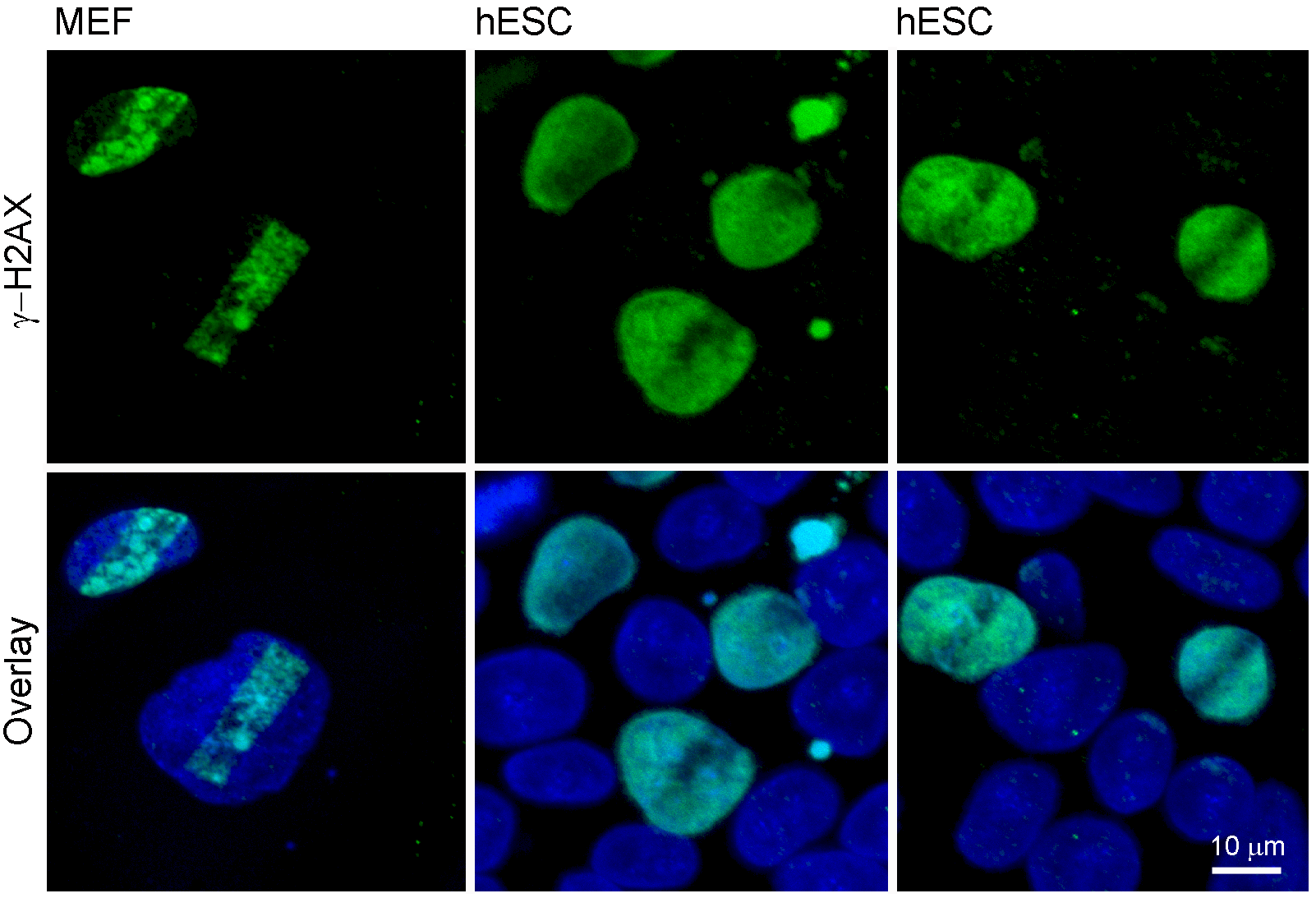

Supplement: Figure S3 — Induction of localized DNA damage in mouse embryonic fibroblasts (MEF) and human embryonic stem cells (hESC). A 405 nm laser was used at 25% intensity to induce DNA damage in defined nuclear region of MEF and hESC. Thirty minutes following DNA damage, cells were fixed and stained for double strand break marker γ-H2AX. Note that only cells affected with laser show γ-H2AX staining. Green - γ-H2AX, Blue - DNA. Scale bar = 10 µm. (1.26 MB TIF) [file pone.0013410.s003.tif]

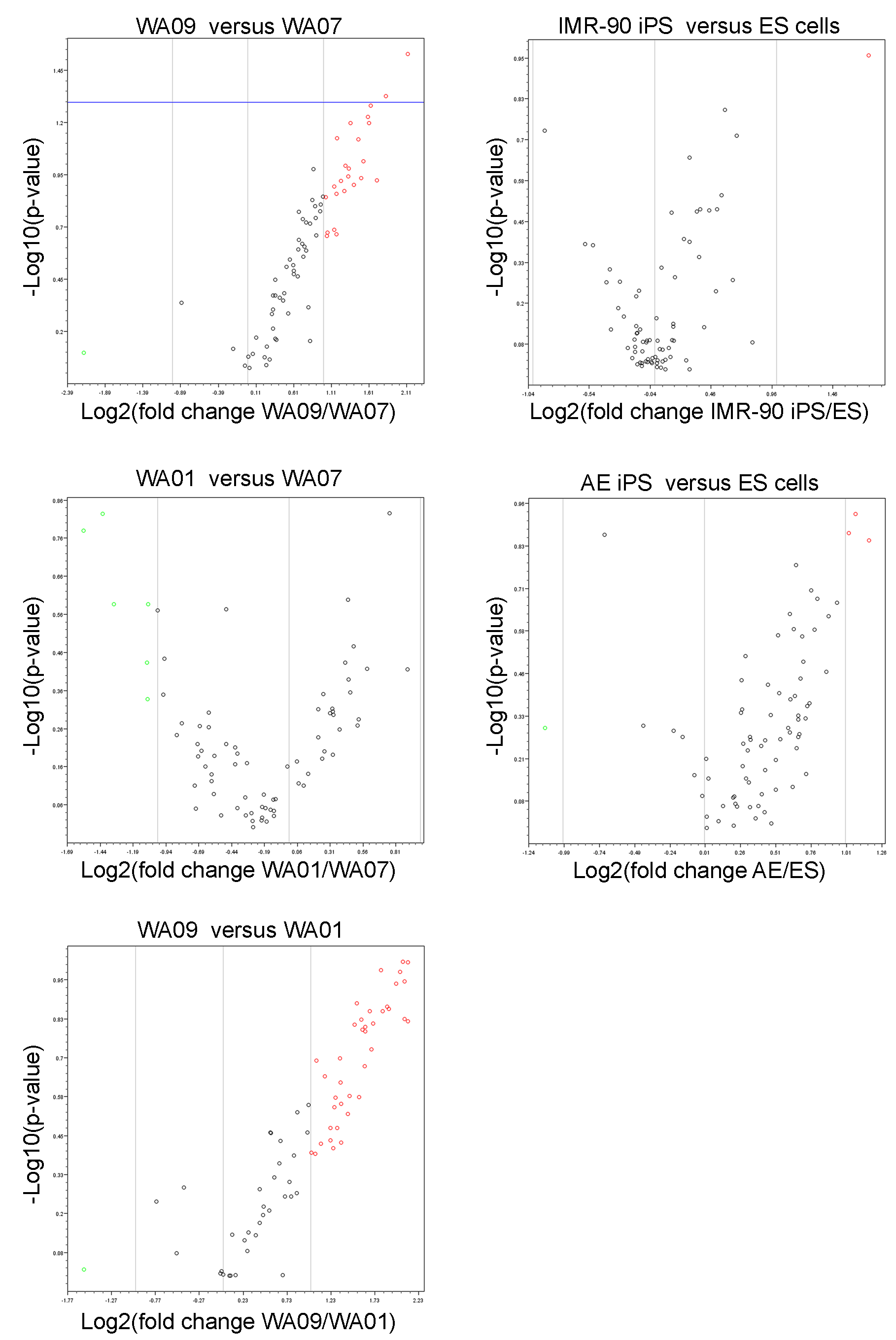

Supplement: Figure S4 — Gene expression comparison between pluripotent stem cell lines. Volcano plots represent p-values (Y-axis) for observed difference in gene expression (X-axis). Blue horizontal line represents position of -Log10(0.05) for easier visualization of significant (p<0.05) difference. Horizontal lines represent two fold boundaries: on the left are genes with more than two fold downregulation (green), and on the right are genes with more than two fold upregulation (red) in comparison to the control line. Genes in between vertical lines show a less than two fold difference in gene expression and are depicted in black. Dots represent genes on the array. (0.32 MB TIF) [file pone.0013410.s004.tif]
