# Supplementary material for: DNA Damage Responses in Human Induced Pluripotent Stem Cells and Embryonic Stem Cells
Source: PLoS One. 2010 Oct 15;5(10):e13410. doi: 10.1371/journal.pone.0013410 (PMC2955528; doi:10.1371/journal.pone.0013410)
Supplement: Table S1 — Detailed analysis of DNA Damage Signaling PCR Array. (0.83 MB DOC) [file pone.0013410.s005.doc]

**Table S**1.

| **A: Layout of DNA Damage Signaling PCR Array catalogue number: PAHS-029.** | | | | | | |
| --- | --- | --- | --- | --- | --- | --- |
| **Position** | **Unigene** | **Refseq** | **Symbol** | **Description** | **Gname** | **RT2 Catalog** |
| A01 | Hs.431048 | NM_005157 | ABL1 | C-abl oncogene 1, receptor tyrosine kinase | ABL/JTK7 | PPH00087E |
| A02 | Hs.601206 | NM_198889 | ANKRD17 | Ankyrin repeat domain 17 | GTAR/NY-BR-16 | PPH12069A |
| A03 | Hs.73722 | NM_080649 | APEX1 | APEX nuclease (multifunctional DNA repair enzyme) 1 | APE/APE1 | PPH02201A |
| A04 | Hs.367437 | NM_000051 | ATM | Ataxia telangiectasia mutated | AT1/ATA | PPH00325B |
| A05 | Hs.271791 | NM_001184 | ATR | Ataxia telangiectasia and Rad3 related | FRP1/MEC1 | PPH01318B |
| A06 | Hs.533526 | NM_000489 | ATRX | Alpha thalassemia/mental retardation syndrome X-linked (RAD54 homolog, S. cerevisiae) | ATR2/MRXHF1 | PPH00470E |
| A07 | Hs.194143 | NM_007294 | BRCA1 | Breast cancer 1, early onset | BRCAI/BRCC1 | PPH00322E |
| A08 | Hs.519162 | NM_006763 | BTG2 | BTG family, member 2 | PC3/TIS21 | PPH01750B |
| A09 | Hs.292524 | NM_001239 | CCNH | Cyclin H | CAK/p34 | PPH00959E |
| A10 | Hs.184298 | NM_001799 | CDK7 | Cyclin-dependent kinase 7 | CAK1/CDKN7 | PPH00935E |
| A11 | Hs.24529 | NM_001274 | CHEK1 | CHK1 checkpoint homolog (S. pombe) | CHK1 | PPH00940B |
| A12 | Hs.291363 | NM_007194 | CHEK2 | CHK2 checkpoint homolog (S. pombe) | CDS1/CHK2 | PPH00921B |
| B01 | Hs.135471 | NM_006384 | CIB1 | Calcium and integrin binding 1 (calmyrin) | CIB/KIP | PPH12320B |
| B02 | Hs.249129 | NM_001279 | CIDEA | Cell death-inducing DFFA-like effector a | CIDE-A | PPH00899B |
| B03 | Hs.151573 | NM_004075 | CRY1 | Cryptochrome 1 (photolyase-like) | PHLL1 | PPH06231B |
| B04 | Hs.290758 | NM_001923 | DDB1 | Damage-specific DNA binding protein 1, 127kDa | DDBA/UV-DDB1 | PPH01515A |
| B05 | Hs.505777 | NM_004083 | DDIT3 | DNA-damage-inducible transcript 3 | CEBPZ/CHOP | PPH00310A |
| B06 | Hs.339396 | NM_007068 | DMC1 | DMC1 dosage suppressor of mck1 homolog, meiosis-specific homologous recombination (yeast) | DMC1H/HsLim15 | PPH02712E |
| B07 | Hs.435981 | NM_001983 | ERCC1 | Excision repair cross-complementing rodent repair deficiency, complementation group 1 (includes overlapping antisense sequence) | COFS4/UV20 | PPH01539A |
| B08 | Hs.487294 | NM_000400 | ERCC2 | Excision repair cross-complementing rodent repair deficiency, complementation group 2 | COFS2/EM9 | PPH01550B |
| B09 | Hs.498248 | NM_130398 | EXO1 | Exonuclease 1 | HEX1/hExoI | PPH02715A |
| B10 | Hs.591084 | NM_004629 | FANCG | Fanconi anemia, complementation group G | FAG/XRCC9 | PPH20387A |
| B11 | Hs.409065 | NM_004111 | FEN1 | Flap structure-specific endonuclease 1 | FEN-1/MF1 | PPH00502A |
| B12 | Hs.292493 | NM_001469 | XRCC6 | X-ray repair complementing defective repair in Chinese hamster cells 6 | CTC75/CTCBF | PPH02175A |
| C01 | Hs.80409 | NM_001924 | GADD45A | Growth arrest and DNA-damage-inducible, alpha | DDIT1/GADD45 | PPH00148B |
| C02 | Hs.9701 | NM_006705 | GADD45G | Growth arrest and DNA-damage-inducible, gamma | CR6/DDIT2 | PPH02207A |
| C03 | Hs.661218 | NM_002066 | GML | Glycosylphosphatidylinositol anchored molecule like protein | LY6DL | PPH01755E |
| C04 | Hs.577202 | NM_005316 | GTF2H1 | General transcription factor IIH, polypeptide 1, 62kDa | BTF2/TFB1 | PPH02600E |
| C05 | Hs.191356 | NM_001515 | GTF2H2 | General transcription factor IIH, polypeptide 2, 44kDa | BTF2/BTF2P44 | PPH02739A |
| C06 | Hs.386189 | NM_016426 | GTSE1 | G-2 and S-phase expressed 1 | B99 | PPH01748E |
| C07 | Hs.152983 | NM_004507 | HUS1 | HUS1 checkpoint homolog (S. pombe) | Hus1 | PPH00922B |
| C08 | Hs.503048 | NM_002180 | IGHMBP2 | Immunoglobulin mu binding protein 2 | CATF1/HCSA | PPH08993E |
| C09 | Hs.17253 | NM_054111 | IP6K3 | Inositol hexakisphosphate kinase 3 | IHPK3/INSP6K3 | PPH08133A |
| C10 | Hs.61188 | NM_033276 | XRCC6BP1 | XRCC6 binding protein 1 | KUB3 | PPH09665E |
| C11 | Hs.1770 | NM_000234 | LIG1 | Ligase I, DNA, ATP-dependent | MGC117397 | PPH02094A |
| C12 | Hs.463978 | NM_002758 | MAP2K6 | Mitogen-activated protein kinase kinase 6 | MAPKK6/MEK6 | PPH00742A |
| D01 | Hs.432642 | NM_002969 | MAPK12 | Mitogen-activated protein kinase 12 | ERK3/ERK6 | PPH01779A |
| D02 | Hs.35947 | NM_003925 | MBD4 | Methyl-CpG binding domain protein 4 | MED1 | PPH02709A |
| D03 | Hs.195364 | NM_000249 | MLH1 | MutL homolog 1, colon cancer, nonpolyposis type 2 (E. coli) | COCA2/FCC2 | PPH00196E |
| D04 | Hs.436650 | NM_014381 | MLH3 | MutL homolog 3 (E. coli) | HNPCC7 | PPH02700A |
| D05 | Hs.509523 | NM_002431 | MNAT1 | Menage a trois homolog 1, cyclin H assembly factor (Xenopus laevis) | MAT1/RNF66 | PPH02714A |
| D06 | Hs.459596 | NM_002434 | MPG | N-methylpurine-DNA glycosylase | AAG/APNG | PPH02102A |
| D07 | Hs.192649 | NM_005590 | MRE11A | MRE11 meiotic recombination 11 homolog A (S. cerevisiae) | ATLD/HNGS1 | PPH01097B |
| D08 | Hs.597656 | NM_000251 | MSH2 | MutS homolog 2, colon cancer, nonpolyposis type 1 (E. coli) | COCA1/FCC1 | PPH00197E |
| D09 | Hs.280987 | NM_002439 | MSH3 | MutS homolog 3 (E. coli) | DUP/MRP1 | PPH02195A |
| D10 | Hs.271353 | NM_012222 | MUTYH | MutY homolog (E. coli) | MYH | PPH02697E |
| D11 | Hs.391463 | NM_018177 | N4BP2 | Nedd4 binding protein 2 | B3BP | PPH19003A |
| D12 | Hs.492208 | NM_002485 | NBN | Nibrin | AT-V1/AT-V2 | PPH00946B |
| E01 | Hs.66196 | NM_002528 | NTHL1 | Nth endonuclease III-like 1 (E. coli) | NTH1/OCTS3 | PPH02720A |
| E02 | Hs.380271 | NM_002542 | OGG1 | 8-oxoguanine DNA glycosylase | HMMH/HOGG1 | PPH02103A |
| E03 | Hs.20930 | NM_020418 | PCBP4 | Poly(rC) binding protein 4 | LIP4/MCG10 | PPH01756B |
| E04 | Hs.147433 | NM_182649 | PCNA | Proliferating cell nuclear antigen | MGC8367 | PPH00216B |
| E05 | Hs.424932 | NM_004208 | AIFM1 | Apoptosis-inducing factor, mitochondrion-associated, 1 | AIF/PDCD8 | PPH01037A |
| E06 | Hs.111749 | NM_000534 | PMS1 | PMS1 postmeiotic segregation increased 1 (S. cerevisiae) | DKFZp781M0253/HNPCC3 | PPH02155A |
| E07 | Hs.632637 | NM_000535 | PMS2 | PMS2 postmeiotic segregation increased 2 (S. cerevisiae) | HNPCC4/PMS2CL | PPH02095E |
| E08 | Hs.225784 | NM_005395 | PMS2L3 | Postmeiotic segregation increased 2-like 3 | PMS2L9/PMS5 | PPH02741B |
| E09 | Hs.78016 | NM_007254 | PNKP | Polynucleotide kinase 3'-phosphatase | PNK | PPH02725A |
| E10 | Hs.631593 | NM_014330 | PPP1R15A | Protein phosphatase 1, regulatory (inhibitor) subunit 15A | GADD34 | PPH02081E |
| E11 | Hs.491682 | NM_006904 | PRKDC | Protein kinase, DNA-activated, catalytic polypeptide | DNA-PKcs/DNAPK | PPH01309B |
| E12 | Hs.531879 | NM_002853 | RAD1 | RAD1 homolog (S. pombe) | HRAD1/REC1 | PPH00741E |
| F01 | Hs.16184 | NM_002873 | RAD17 | RAD17 homolog (S. pombe) | CCYC/HRAD17 | PPH00929E |
| F02 | Hs.375684 | NM_020165 | RAD18 | RAD18 homolog (S. cerevisiae) | RNF73 | PPH02740A |
| F03 | Hs.81848 | NM_006265 | RAD21 | RAD21 homolog (S. pombe) | HR21/HRAD21 | PPH10216A |
| F04 | Hs.655835 | NM_005732 | RAD50 | RAD50 homolog (S. cerevisiae) | RAD50-2/hRad50 | PPH00956A |
| F05 | Hs.631709 | NM_002875 | RAD51 | RAD51 homolog (RecA homolog, E. coli) (S. cerevisiae) | BRCC5/HRAD51 | PPH00942E |
| F06 | Hs.172587 | NM_133509 | RAD51L1 | RAD51-like 1 (S. cerevisiae) | R51H2/RAD51B | PPH02654B |
| F07 | Hs.655354 | NM_004584 | RAD9A | RAD9 homolog A (S. pombe) | RAD9 | PPH00944B |
| F08 | Hs.546282 | NM_002894 | RBBP8 | Retinoblastoma binding protein 8 | CTIP/RIM | PPH00954E |
| F09 | Hs.443077 | NM_016316 | REV1 | REV1 homolog (S. cerevisiae) | REV1L | PPH21080A |
| F10 | Hs.461925 | NM_002945 | RPA1 | Replication protein A1, 70kDa | HSSB/MST075 | PPH02730A |
| F11 | Hs.408846 | NM_022367 | SEMA4A | Sema domain, immunoglobulin domain (Ig), transmembrane domain (TM) and short cytoplasmic domain, (semaphorin) 4A | CORD10/RP35 | PPH19432A |
| F12 | Hs.591336 | NM_014454 | SESN1 | Sestrin 1 | PA26/SEST1 | PPH01759B |
| G01 | Hs.211602 | NM_006306 | SMC1A | Structural maintenance of chromosomes 1A | CDLS2/DKFZp686L19178 | PPH14489A |
| G02 | Hs.81424 | NM_003352 | SUMO1 | SMT3 suppressor of mif two 3 homolog 1 (S. cerevisiae) | DAP-1/GMP1 | PPH00973E |
| G03 | Hs.654481 | NM_000546 | TP53 | Tumor protein p53 | LFS1/TRP53 | PPH00213E |
| G04 | Hs.697294 | NM_005427 | TP73 | Tumor protein p73 | P73 | PPH00725A |
| G05 | Hs.707026 | NM_016381 | TREX1 | Three prime repair exonuclease 1 | AGS1/AGS5 | PPH02340B |
| G06 | Hs.191334 | NM_003362 | UNG | Uracil-DNA glycosylase | DGU/DKFZp781L1143 | PPH01727E |
| G07 | Hs.654364 | NM_000380 | XPA | Xeroderma pigmentosum, complementation group A | XP1/XPAC | PPH01524B |
| G08 | Hs.475538 | NM_004628 | XPC | Xeroderma pigmentosum, complementation group C | XP3/XPCC | PPH01536E |
| G09 | Hs.98493 | NM_006297 | XRCC1 | X-ray repair complementing defective repair in Chinese hamster cells 1 | RCC | PPH01741A |
| G10 | Hs.647093 | NM_005431 | XRCC2 | X-ray repair complementing defective repair in Chinese hamster cells 2 | DKFZp781P0919 | PPH01694A |
| G11 | Hs.592325 | NM_005432 | XRCC3 | X-ray repair complementing defective repair in Chinese hamster cells 3 | XRCC3 | PPH02208A |
| G12 | Hs.444451 | NM_016653 | ZAK | Sterile alpha motif and leucine zipper containing kinase AZK | AZK/MLK7 | PPH05541E |
| H01 | Hs.534255 | NM_004048 | B2M | Beta-2-microglobulin | B2M | PPH01094E |
| H02 | Hs.412707 | NM_000194 | HPRT1 | Hypoxanthine phosphoribosyltransferase 1 | HGPRT/HPRT | PPH01018B |
| H03 | Hs.523185 | NM_012423 | RPL13A | Ribosomal protein L13a | RPL13A | PPH01020B |
| H04 | Hs.592355 | NM_002046 | GAPDH | Glyceraldehyde-3-phosphate dehydrogenase | G3PD/GAPD | PPH00150E |
| H05 | Hs.520640 | NM_001101 | ACTB | Actin, beta | PS1TP5BP1 | PPH00073E |
| H06 | N/A | SA_00105 | HGDC | Human Genomic DNA Contamination | HIGX1A |  |
| H07 | N/A | SA_00104 | RTC | Reverse Transcription Control | RTC |  |
| H08 | N/A | SA_00104 | RTC | Reverse Transcription Control | RTC |  |
| H09 | N/A | SA_00104 | RTC | Reverse Transcription Control | RTC |  |
| H10 | N/A | SA_00103 | PPC | Positive PCR Control | PPC |  |
| H11 | N/A | SA_00103 | PPC | Positive PCR Control | PPC |  |
| H12 | N/A | SA_00103 | PPC | Positive PCR Control | PPC |  |

**B: Average Ct value and Standard deviations for each group.**

| **Ct values** | | | | | | | **Standard Deviation** | | | | |
| --- | --- | --- | --- | --- | --- | --- | --- | --- | --- | --- | --- |
| **Well** | **Symbol** | **IMR-90 iPS** | **IMR-90** | **TF** | **AE iPS** | **Control Group (ES)** | **IMR-90 iPS** | **IMR-90** | **TF** | **AE iPS** | **Control Group (ES)** |
| A01 | ABL1 | 23.69 | 23.18 | 24.35 | 23.97 | 23.69 | 0.201647 | 0.418426 | 0.677464 | 0.489414 | 0.404731 |
| A02 | ANKRD17 | 22.76 | 23.71 | 23.79 | 23.28 | 22.87 | 0.40777 | 0.428733 | 0.639084 | 0.848612 | 0.427662 |
| A03 | APEX1 | 21.03 | 23.14 | 21.98 | 20.68 | 20.9 | 0.315776 | 0.873483 | 0.736414 | 0.610756 | 0.378795 |
| A04 | ATM | 22.76 | 24.67 | 23.86 | 25.14 | 23.29 | 2.476369 | 1.417436 | 2.09902 | 3.231355 | 2.547224 |
| A05 | ATR | 22.54 | 24.87 | 23.84 | 24.29 | 22.86 | 1.180641 | 0.681506 | 0.92696 | 1.947989 | 1.313542 |
| A06 | ATRX | 26.45 | 27.37 | 28.13 | 26.53 | 26.19 | 0.099939 | 0.270499 | 1.099648 | 0.83567 | 0.637851 |
| A07 | BRCA1 | 24.8 | 28.17 | 26.83 | 25.35 | 24.84 | 0.339502 | 0.78598 | 0.914173 | 0.950398 | 0.459084 |
| A08 | BTG2 | 29.08 | 29.4 | 28.7 | 31.15 | 29.29 | 1.424588 | 0.631434 | 0.407057 | 1.470364 | 1.172487 |
| A09 | CCNH | 23.01 | 23.76 | 23.08 | 23.07 | 23.1 | 0.270719 | 0.357315 | 0.197648 | 0.329027 | 0.552217 |
| A10 | CDK7 | 22.46 | 22.27 | 22.46 | 22.4 | 22.46 | 0.08715 | 0.18673 | 0.402924 | 0.18397 | 0.491124 |
| A11 | CHEK1 | 21.95 | 25.44 | 24.45 | 21.68 | 22.03 | 0.103495 | 0.689791 | 0.666404 | 0.08789 | 0.425082 |
| A12 | CHEK2 | 24.07 | 27.41 | 27.05 | 24.37 | 23.87 | 0.339167 | 0.398519 | 0.379266 | 0.728572 | 0.688454 |
| B01 | CIB1 | 23.63 | 22.46 | 22.02 | 23.43 | 23.47 | 0.377224 | 0.427314 | 0.629831 | 0.314316 | 0.331247 |
| B02 | CIDEA | 34.57 | 33.59 | 31.43 | 34.1 | 33.68 | 0.776116 | 1.352846 | 0.323774 | 0.785594 | 1.3985 |
| B03 | CRY1 | 23.37 | 24.28 | 24.53 | 23.67 | 23.38 | 0.235798 | 0.563322 | 0.359584 | 0.39566 | 0.563184 |
| B04 | DDB1 | 22.09 | 22.62 | 22.95 | 22.74 | 22.28 | 0.412741 | 0.571714 | 0.119441 | 0.872951 | 0.869355 |
| B05 | DDIT3 | 23.79 | 24.02 | 22.8 | 25.13 | 25.58 | 0.970393 | 0.989024 | 0.726384 | 0.603323 | 0.800508 |
| B06 | DMC1 | 28.3 | 33.73 | 30.48 | 28.46 | 27.73 | 0.225026 | 0.959638 | 1.233053 | 0.341794 | 0.715369 |
| B07 | ERCC1 | 25.14 | 24.86 | 24.59 | 25.76 | 25.31 | 0.678714 | 0.716692 | 0.416161 | 1.119095 | 0.728854 |
| B08 | ERCC2 | 23.87 | 25.19 | 25.06 | 24.69 | 24.27 | 0.310854 | 0.493689 | 0.154456 | 1.152842 | 0.775289 |
| B09 | EXO1 | 24.18 | 27.4 | 26.51 | 23.95 | 23.84 | 0.199567 | 1.256941 | 0.823892 | 0.472973 | 0.43702 |
| B10 | FANCG | 24.44 | 26.95 | 26.47 | 24.85 | 24.41 | 0.303436 | 0.491169 | 0.515225 | 0.713315 | 0.564024 |
| B11 | FEN1 | 22.85 | 25.97 | 25.09 | 23.55 | 23.12 | 0.361073 | 0.961774 | 0.657458 | 1.037922 | 0.894428 |
| B12 | XRCC6 | 21.98 | 23.98 | 23.45 | 22.38 | 21.93 | 0.146197 | 0.439591 | 0.340144 | 0.193825 | 0.397864 |
| C01 | GADD45A | 26.4 | 26.01 | 25.23 | 27.47 | 26.77 | 0.725203 | 1.080011 | 0.481113 | 0.578038 | 0.661154 |
| C02 | GADD45G | 26.31 | 31.13 | 30.86 | 27.91 | 27.95 | 1.324409 | 1.08048 | 1.003476 | 0.935364 | 1.56702 |
| C03 | GML | 34.38 | 35 | 35 | 35 | 34.21 | 1.232305 | 0 | 0 | 0 | 1.982402 |
| C04 | GTF2H1 | 24.2 | 24.13 | 22.91 | 23.95 | 24.1 | 0.079431 | 0.432113 | 0.433734 | 0.191344 | 0.404911 |
| C05 | GTF2H2 | 22.26 | 24.06 | 23.98 | 23.2 | 22.69 | 0.298952 | 0.368765 | 0.593011 | 0.369463 | 0.788665 |
| C06 | GTSE1 | 25.08 | 28.1 | 27.43 | 24.88 | 24.8 | 0.139571 | 0.712845 | 2.300637 | 0.339951 | 0.401958 |
| C07 | HUS1 | 25.49 | 27.07 | 26.28 | 26 | 25.37 | 0.149309 | 1.129849 | 0.358295 | 0.93408 | 0.667748 |
| C08 | IGHMBP2 | 25.47 | 26.32 | 27.28 | 26.36 | 26.17 | 0.19154 | 0.334414 | 0.131592 | 1.031572 | 0.571904 |
| C09 | IP6K3 | 30.98 | 31.79 | 31.04 | 31.64 | 30.75 | 1.016591 | 2.163841 | 2.950842 | 1.405278 | 1.371141 |
| C10 | XRCC6BP1 | 26.75 | 28.45 | 27.19 | 26.54 | 26.42 | 0.38346 | 0.135656 | 0.329824 | 0.304443 | 0.63256 |
| C11 | LIG1 | 25.17 | 28.3 | 27.42 | 25.53 | 25.14 | 0.27887 | 0.777872 | 0.716207 | 1.017541 | 0.656065 |
| C12 | MAP2K6 | 24.38 | 28.11 | 26.27 | 23.71 | 23.5 | 1.260547 | 0.548785 | 0.940633 | 0.185997 | 0.290311 |
| D01 | MAPK12 | 25.32 | 24.42 | 24.59 | 26.37 | 25.63 | 0.280093 | 0.346576 | 0.23731 | 1.22689 | 0.750614 |
| D02 | MBD4 | 22.75 | 23.14 | 23.71 | 22.71 | 22.67 | 0.177495 | 0.294923 | 0.139122 | 0.217278 | 0.36616 |
| D03 | MLH1 | 22.71 | 24.05 | 23.51 | 22.29 | 22.23 | 0.19393 | 0.39593 | 0.341313 | 0.204522 | 0.377139 |
| D04 | MLH3 | 25.19 | 26.54 | 26.7 | 25.74 | 25.5 | 0.189214 | 0.234362 | 0.994356 | 0.796868 | 0.593385 |
| D05 | MNAT1 | 23.15 | 23.78 | 23.51 | 23.25 | 23.19 | 0.217328 | 0.369868 | 0.273551 | 0.214332 | 0.675539 |
| D06 | MPG | 23.63 | 22.6 | 22.58 | 24.17 | 23.71 | 0.430522 | 0.235091 | 0.31954 | 0.535681 | 0.543166 |
| D07 | MRE11A | 26.31 | 27.78 | 27.93 | 26.7 | 25.76 | 0.127848 | 0.297472 | 0.394557 | 1.072756 | 0.645287 |
| D08 | MSH2 | 20.39 | 24.98 | 23.91 | 20.53 | 20.53 | 0.242628 | 0.414147 | 0.40864 | 0.547074 | 0.587079 |
| D09 | MSH3 | 24.44 | 25.52 | 24.98 | 24.73 | 24.53 | 0.336636 | 0.243677 | 0.397407 | 0.816631 | 0.511559 |
| D10 | MUTYH | 24.3 | 27.5 | 27.11 | 24.78 | 24.69 | 0.144795 | 0.588666 | 0.428355 | 0.63162 | 0.488911 |
| D11 | N4BP2 | 23.74 | 27.08 | 26.41 | 24.1 | 23.8 | 0.318312 | 0.804577 | 0.872001 | 0.800265 | 0.618942 |
| D12 | NBN | 25.82 | 27.56 | 26.28 | 26.43 | 25.74 | 0.284865 | 0.499093 | 0.35231 | 0.79426 | 0.61967 |
| E01 | NTHL1 | 23.45 | 25.73 | 25.47 | 23.33 | 23.63 | 0.166243 | 0.295551 | 0.735117 | 0.47913 | 0.31938 |
| E02 | OGG1 | 24.08 | 25.06 | 24.56 | 24.36 | 24.04 | 0.163694 | 0.367594 | 0.337317 | 0.586042 | 0.33062 |
| E03 | PCBP4 | 30.62 | 29.66 | 29.92 | 32.81 | 31.58 | 0.514211 | 0.932966 | 0.19137 | 1.659804 | 1.430867 |
| E04 | PCNA | 20.21 | 22.7 | 21.9 | 20.19 | 20.08 | 0.086645 | 0.687798 | 0.71686 | 0.304727 | 0.379319 |
| E05 | AIFM1 | 22.86 | 25.61 | 23.96 | 24.12 | 23.53 | 0.175063 | 0.263904 | 0.187212 | 0.196345 | 0.77474 |
| E06 | PMS1 | 22.55 | 24.9 | 24.44 | 22.93 | 22.83 | 0.129178 | 0.1087 | 0.473285 | 0.634621 | 0.440947 |
| E07 | PMS2 | 24.18 | 24.74 | 24.96 | 24.87 | 24.76 | 0.258806 | 0.309391 | 0.459628 | 0.916056 | 0.731148 |
| E08 | PMS2L3 | 23.17 | 23.22 | 24.12 | 23.75 | 23.35 | 0.235477 | 0.589082 | 0.995791 | 0.793782 | 0.51509 |
| E09 | PNKP | 24.19 | 25.53 | 25.38 | 24.97 | 24.72 | 0.447733 | 0.571166 | 0.531201 | 1.49499 | 0.775224 |
| E10 | PPP1R15A | 26.4 | 24.74 | 24.7 | 28.15 | 27 | 1.079622 | 0.385176 | 0.424858 | 1.082383 | 0.848359 |
| E11 | PRKDC | 21.07 | 23.38 | 23.01 | 21.79 | 21 | 0.389903 | 0.468351 | 0.526203 | 0.804079 | 0.40021 |
| E12 | RAD1 | 24.52 | 24.92 | 25.94 | 24.45 | 24.4 | 0.213742 | 0.283406 | 0.451065 | 0.17432 | 0.428872 |
| F01 | RAD17 | 23.39 | 24.65 | 24.19 | 23.31 | 23.31 | 0.364952 | 0.370638 | 0.719881 | 0.175423 | 0.440777 |
| F02 | RAD18 | 23.43 | 25.34 | 24.72 | 23.72 | 23.54 | 0.226319 | 0.499614 | 0.342699 | 0.555229 | 0.507248 |
| F03 | RAD21 | 20.5 | 22.32 | 21.62 | 20.97 | 20.65 | 0.159049 | 0.405191 | 0.280462 | 0.51914 | 0.345069 |
| F04 | RAD50 | 26.22 | 27.51 | 26.91 | 27.47 | 27.05 | 0.30567 | 0.468994 | 0.568606 | 2.00707 | 2.842825 |
| F05 | RAD51 | 28.63 | 30.52 | 30.08 | 28.21 | 28.03 | 0.342763 | 0.438787 | 0.261583 | 0.42536 | 0.537924 |
| F06 | RAD51L1 | 24.63 | 26.18 | 25.52 | 25.19 | 24.67 | 0.21187 | 0.160388 | 0.448218 | 0.928233 | 0.607291 |
| F07 | RAD9A | 26.27 | 27.86 | 27.65 | 26.54 | 26.14 | 0.535294 | 0.437913 | 0.458456 | 0.590852 | 0.633239 |
| F08 | RBBP8 | 22.66 | 24.66 | 24.04 | 22.47 | 22.61 | 0.234921 | 0.541815 | 0.75435 | 0.232588 | 0.41787 |
| F09 | REV1 | 24.44 | 25.43 | 25.53 | 24.56 | 24.49 | 0.178366 | 0.395463 | 0.906293 | 0.566167 | 0.59015 |
| F10 | RPA1 | 22.24 | 24.03 | 23.77 | 22.98 | 22.11 | 0.321382 | 0.362588 | 0.177874 | 1.105261 | 0.439163 |
| F11 | SEMA4A | 28.93 | 31.23 | 33.32 | 29.4 | 29.21 | 0.522504 | 1.029461 | 1.089592 | 1.927212 | 1.354465 |
| F12 | SESN1 | 24.18 | 26.35 | 25.39 | 24.84 | 24.35 | 0.47909 | 0.705535 | 0.209073 | 0.633859 | 0.736635 |
| G01 | SMC1A | 22.32 | 24.13 | 24.33 | 22.52 | 22.5 | 0.297289 | 0.727107 | 0.44288 | 1.12266 | 0.663619 |
| G02 | SUMO1 | 21.61 | 22.97 | 22.65 | 21.68 | 21.66 | 0.187572 | 0.482518 | 0.235579 | 0.28445 | 0.507761 |
| G03 | TP53 | 21.48 | 23.4 | 22.69 | 21.62 | 21.35 | 0.235418 | 0.627407 | 0.191993 | 0.503912 | 0.565228 |
| G04 | TP73 | 32 | 35 | 35 | 33.22 | 33.16 | 1.109547 | 0 | 0 | 1.05355 | 1.600998 |
| G05 | TREX1 | 27.47 | 26.46 | 25.56 | 27.55 | 27.5 | 0.307623 | 0.657722 | 0.403084 | 0.703812 | 0.666074 |
| G06 | UNG | 21.63 | 25.22 | 24.47 | 21.19 | 21.26 | 0.151274 | 0.235897 | 0.305618 | 0.140817 | 0.337884 |
| G07 | XPA | 25.14 | 24.91 | 25.01 | 25.41 | 25.05 | 0.405685 | 0.283134 | 0.480011 | 0.42175 | 0.651178 |
| G08 | XPC | 24.03 | 24.56 | 24.05 | 23.88 | 23.88 | 0.359445 | 0.220837 | 0.645187 | 0.414078 | 0.496499 |
| G09 | XRCC1 | 24.64 | 25.93 | 25.63 | 24.69 | 24.62 | 0.107263 | 0.541068 | 0.215122 | 0.728733 | 0.410277 |
| G10 | XRCC2 | 24.07 | 28.31 | 27.46 | 24.18 | 23.97 | 0.449718 | 1.06517 | 0.844579 | 1.131949 | 0.623557 |
| G11 | XRCC3 | 28.62 | 28.82 | 29.77 | 29.79 | 29.09 | 0.586141 | 0.76989 | 0.903079 | 1.76152 | 1.195661 |
| G12 | ZAK | 25.78 | 22.54 | 23.79 | 26.26 | 25.55 | 0.107746 | 0.286064 | 0.287941 | 0.842753 | 0.86414 |
| H01 | B2M | 22.23 | 19.09 | 18.75 | 22.61 | 22.24 | 0.35918 | 0.282999 | 0.569137 | 0.289155 | 0.61333 |
| H02 | HPRT1 | 21.31 | 23.59 | 22.12 | 21.94 | 21.81 | 0.210848 | 0.576289 | 0.344069 | 0.169098 | 0.599773 |
| H03 | RPL13A | 18.45 | 19.28 | 18.94 | 18.66 | 18.81 | 0.20909 | 0.101719 | 0.450428 | 0.198478 | 0.503662 |
| H04 | GAPDH | 17.15 | 16.65 | 17.39 | 16.86 | 17.1 | 0.275119 | 0.518097 | 0.510724 | 0.434447 | 0.453925 |
| H05 | ACTB | 16.42 | 16.45 | 16.39 | 17.17 | 16.45 | 0.981147 | 0.292848 | 0.966319 | 1.169646 | 1.023321 |
| H06 | HGDC | 31.91 | 32.15 | 31.79 | 33.9 | 31.59 | 1.918776 | 2.070562 | 2.814781 | 1.899315 | 1.974006 |
| H07 | RTC | 22.46 | 22.4 | 22.38 | 22.22 | 22.22 | 0.730175 | 0.607225 | 0.643535 | 0.616447 | 0.565481 |
| H08 | RTC | 22.45 | 22.36 | 22.39 | 22.04 | 22.26 | 0.677945 | 0.594438 | 0.676328 | 0.486014 | 0.569184 |
| H09 | RTC | 22.47 | 22.4 | 22.42 | 22.12 | 22.36 | 0.703234 | 0.563781 | 0.646171 | 0.567335 | 0.512 |
| H10 | PPC | 19.3 | 19.29 | 19.35 | 19.59 | 19.08 | 0.086271 | 0.121897 | 0.153675 | 0.270519 | 0.287168 |
| H11 | PPC | 19.38 | 19.18 | 19.44 | 19.72 | 19.25 | 0.072903 | 0.351536 | 0.177215 | 0.525076 | 0.207487 |
| H12 | PPC | 19.39 | 19.3 | 19.33 | 19.26 | 19.21 | 0.115105 | 0.043171 | 0.180886 | 0.340937 | 0.241197 |

**C: Average Delta(Ct) values and standard deviation for each group.**

**Average Delta (Ct) = Ct(GOI) - Ave Ct(HKG)** Standard Deviation

| **Well** | **Symbol** | **IMR-90 iPS** | **IMR-90** | **TF** | **AE iPS** | **Control Group (ES)** | **IMR-90 iPS** | **IMR-90** | **TF** | **AE iPS** | **Control Group (ES)** |
| --- | --- | --- | --- | --- | --- | --- | --- | --- | --- | --- | --- |
| A01 | ABL1 | 7.265348 | 6.738962 | 7.958836 | 6.807852 | 7.239069 | 0.864373 | 0.371215 | 1.408534 | 0.789778 | 0.848983 |
| A02 | ANKRD17 | 6.338756 | 7.267981 | 7.403486 | 6.110563 | 6.426949 | 0.687874 | 0.39344 | 1.209951 | 0.327758 | 0.763777 |
| A03 | APEX1 | 4.611145 | 6.69419 | 5.589094 | 3.510914 | 4.450413 | 1.294965 | 1.100943 | 1.696025 | 1.44014 | 1.231459 |
| A04 | ATM | 6.341489 | 8.225868 | 7.470611 | 7.973477 | 6.844369 | 1.519923 | 1.124865 | 1.205032 | 2.182955 | 1.586975 |
| A05 | ATR | 6.121157 | 8.422927 | 7.453601 | 7.11884 | 6.410762 | 0.215015 | 0.407975 | 0.434225 | 0.919344 | 0.472132 |
| A06 | ATRX | 10.026758 | 10.928082 | 11.747043 | 9.360573 | 9.743675 | 1.00499 | 0.140969 | 1.492747 | 0.409221 | 1.38159 |
| A07 | BRCA1 | 8.375734 | 11.721564 | 10.447624 | 8.180187 | 8.392916 | 0.671454 | 0.956546 | 1.80786 | 0.289465 | 1.171512 |
| A08 | BTG2 | 12.658919 | 12.950442 | 12.315595 | 13.978682 | 12.840175 | 2.37293 | 0.868881 | 1.157305 | 0.430645 | 1.536827 |
| A09 | CCNH | 6.584928 | 7.313404 | 6.695947 | 5.906159 | 6.649938 | 1.153809 | 0.472563 | 1.141729 | 0.947792 | 1.204153 |
| A10 | CDK7 | 6.036008 | 5.826995 | 6.070129 | 5.233423 | 6.012716 | 1.015114 | 0.272346 | 0.685669 | 0.992029 | 1.050292 |
| A11 | CHEK1 | 5.531471 | 8.998702 | 8.064471 | 4.51128 | 5.582891 | 0.996325 | 0.893388 | 1.628941 | 1.257517 | 1.094798 |
| A12 | CHEK2 | 7.643335 | 10.959987 | 10.662352 | 7.204292 | 7.424318 | 1.26625 | 0.571881 | 1.343758 | 0.456229 | 1.1444 |
| B01 | CIB1 | 7.208059 | 6.017541 | 5.636272 | 6.267094 | 7.023571 | 1.351588 | 0.684388 | 1.551094 | 1.402507 | 0.992657 |
| B02 | CIDEA | 18.143623 | 17.143829 | 15.03985 | 16.93555 | 17.231431 | 1.748864 | 1.54525 | 1.144682 | 0.762428 | 2.042465 |
| B03 | CRY1 | 6.948959 | 7.836621 | 8.145205 | 6.506194 | 6.931474 | 1.153354 | 0.436288 | 0.813786 | 0.78386 | 1.262016 |
| B04 | DDB1 | 5.671277 | 6.177168 | 6.566169 | 5.572343 | 5.837406 | 0.814865 | 0.549961 | 0.912886 | 0.733178 | 0.520463 |
| B05 | DDIT3 | 7.370418 | 7.578849 | 6.417813 | 7.963061 | 9.129247 | 1.944005 | 1.246303 | 1.658594 | 1.723864 | 1.366055 |
| B06 | DMC1 | 11.872536 | 17.282084 | 14.093531 | 11.289769 | 11.279271 | 1.021464 | 1.158466 | 2.036668 | 1.166769 | 1.271815 |
| B07 | ERCC1 | 8.719194 | 8.412218 | 8.203117 | 8.595955 | 8.86016 | 1.655713 | 0.869663 | 1.382303 | 1.448109 | 0.678074 |
| B08 | ERCC2 | 7.450602 | 8.746331 | 8.669141 | 7.519167 | 7.827874 | 1.157262 | 0.594154 | 1.090096 | 0.715406 | 0.639843 |
| B09 | EXO1 | 7.76118 | 10.956225 | 10.121859 | 6.786892 | 7.393097 | 0.840762 | 1.391879 | 1.730573 | 0.696799 | 1.024181 |
| B10 | FANCG | 8.016556 | 10.508345 | 10.084912 | 7.685248 | 7.958961 | 0.734238 | 0.575537 | 1.456123 | 0.741281 | 0.623736 |
| B11 | FEN1 | 6.426931 | 9.523862 | 8.706931 | 6.380537 | 6.670489 | 0.743263 | 1.009951 | 1.430744 | 0.989235 | 0.486921 |
| B12 | XRCC6 | 5.558017 | 7.535064 | 7.064834 | 5.214694 | 5.48608 | 0.978776 | 0.469771 | 1.180811 | 1.122798 | 0.911157 |
| C01 | GADD45A | 9.974522 | 9.565503 | 8.846868 | 10.306605 | 10.321394 | 1.610948 | 1.248096 | 1.305594 | 1.383538 | 1.029324 |
| C02 | GADD45G | 9.88553 | 14.684801 | 14.4687 | 10.743732 | 11.502193 | 2.291331 | 1.0377 | 1.340707 | 0.863876 | 1.36072 |
| C03 | GML | 17.960583 | 18.554919 | 18.612993 | 17.833517 | 17.767006 | 1.279781 | 0.292848 | 0.966319 | 1.169646 | 2.618362 |
| C04 | GTF2H1 | 7.773569 | 7.687934 | 6.526024 | 6.778634 | 7.65686 | 1.001431 | 0.694768 | 1.374112 | 1.360125 | 1.167261 |
| C05 | GTF2H2 | 5.833946 | 7.616738 | 7.593664 | 6.036539 | 6.242703 | 0.697616 | 0.28504 | 1.24567 | 0.864576 | 1.148267 |
| C06 | GTSE1 | 8.656605 | 11.653225 | 11.047581 | 7.717424 | 8.356983 | 1.102557 | 0.939587 | 2.969726 | 1.498799 | 1.190543 |
| C07 | HUS1 | 9.070158 | 10.620242 | 9.891536 | 8.829434 | 8.928483 | 1.024889 | 1.28069 | 1.297798 | 0.781986 | 0.784039 |
| C08 | IGHMBP2 | 9.050301 | 9.875723 | 10.891169 | 9.196361 | 9.72599 | 1.068255 | 0.305266 | 1.067936 | 0.583516 | 0.718842 |
| C09 | IP6K3 | 14.560537 | 15.348977 | 14.65315 | 14.472102 | 14.304813 | 1.973391 | 1.901253 | 2.045991 | 1.039291 | 1.387963 |
| C10 | XRCC6BP1 | 10.326527 | 12.002069 | 10.804711 | 9.376228 | 9.969193 | 1.331396 | 0.366306 | 1.292278 | 1.214287 | 1.185395 |
| C11 | LIG1 | 8.743328 | 11.858515 | 11.029885 | 8.365539 | 8.691307 | 0.974419 | 0.883491 | 1.611235 | 0.891236 | 0.718811 |
| C12 | MAP2K6 | 7.955725 | 11.664706 | 9.880829 | 6.548377 | 7.052996 | 0.290755 | 0.458024 | 0.613862 | 1.024865 | 1.087895 |
| D01 | MAPK12 | 8.899047 | 7.976519 | 8.202176 | 9.201497 | 9.186748 | 1.1884 | 0.379163 | 1.170401 | 0.560064 | 0.799942 |
| D02 | MBD4 | 6.325064 | 6.698336 | 7.321468 | 5.545142 | 6.225736 | 1.037943 | 0.315315 | 1.102433 | 0.958953 | 1.035923 |
| D03 | MLH1 | 6.291353 | 7.601488 | 7.118892 | 5.121198 | 5.785703 | 0.944339 | 0.608164 | 1.30614 | 1.066135 | 1.144743 |
| D04 | MLH3 | 8.765043 | 10.091384 | 10.314825 | 8.578437 | 9.052148 | 0.920937 | 0.316857 | 1.788245 | 0.380704 | 1.347619 |
| D05 | MNAT1 | 6.725643 | 7.339907 | 7.120319 | 6.08019 | 6.744715 | 1.192574 | 0.635238 | 1.239815 | 0.978361 | 1.218837 |
| D06 | MPG | 7.211535 | 6.152817 | 6.189169 | 7.006539 | 7.26693 | 1.409095 | 0.375699 | 1.285827 | 1.28641 | 0.753213 |
| D07 | MRE11A | 9.885055 | 11.338928 | 11.545676 | 9.533334 | 9.31515 | 0.986536 | 0.294097 | 1.23614 | 0.472548 | 0.839244 |
| D08 | MSH2 | 3.969349 | 8.531847 | 7.520726 | 3.358871 | 4.087132 | 1.130179 | 0.659202 | 1.31661 | 0.735058 | 1.151598 |
| D09 | MSH3 | 8.019992 | 9.071529 | 8.5924 | 7.564582 | 8.084824 | 0.696671 | 0.212504 | 1.029904 | 0.40989 | 0.668261 |
| D10 | MUTYH | 7.87797 | 11.05653 | 10.718113 | 7.611687 | 8.243403 | 0.927934 | 0.541122 | 1.386931 | 0.852464 | 0.765842 |
| D11 | N4BP2 | 7.316209 | 10.638691 | 10.025897 | 6.928594 | 7.358585 | 0.682686 | 0.580394 | 1.260403 | 0.429273 | 0.976916 |
| D12 | NBN | 9.393971 | 11.114482 | 9.892776 | 9.262353 | 9.2914 | 1.137453 | 0.57722 | 1.31431 | 0.378903 | 1.01776 |
| E01 | NTHL1 | 7.029896 | 9.287215 | 9.085975 | 6.164042 | 7.187419 | 1.146659 | 0.438247 | 1.615043 | 1.32877 | 1.113157 |
| E02 | OGG1 | 7.654169 | 8.614983 | 8.176363 | 7.189454 | 7.591485 | 0.83816 | 0.458978 | 1.223362 | 1.070502 | 0.959428 |
| E03 | PCBP4 | 14.201584 | 13.216964 | 13.532807 | 15.643234 | 15.135079 | 1.06471 | 1.085382 | 1.157681 | 1.200381 | 1.076106 |
| E04 | PCNA | 3.791306 | 6.251907 | 5.514657 | 3.024796 | 3.631351 | 0.964768 | 0.834341 | 1.544107 | 1.295322 | 0.85744 |
| E05 | AIFM1 | 6.439491 | 9.166669 | 7.571562 | 6.950099 | 7.082233 | 0.949506 | 0.339199 | 1.136077 | 0.977359 | 1.305592 |
| E06 | PMS1 | 6.130387 | 8.459261 | 8.048951 | 5.763688 | 6.388568 | 0.941496 | 0.208788 | 1.197914 | 0.559805 | 1.234051 |
| E07 | PMS2 | 7.759672 | 8.296148 | 8.576672 | 7.70271 | 8.31204 | 1.147903 | 0.224987 | 1.32123 | 0.501477 | 0.973887 |
| E08 | PMS2L3 | 6.749637 | 6.776949 | 7.736971 | 6.581445 | 6.905033 | 1.02205 | 0.332987 | 1.647487 | 0.387684 | 0.961367 |
| E09 | PNKP | 7.765863 | 9.082567 | 8.995493 | 7.807032 | 8.277137 | 1.325328 | 0.522773 | 1.49515 | 1.15563 | 0.875305 |
| E10 | PPP1R15A | 9.976225 | 8.299731 | 8.317757 | 10.985196 | 10.553321 | 2.031748 | 0.469101 | 1.377871 | 0.988918 | 0.537189 |
| E11 | PRKDC | 4.646021 | 6.931548 | 6.620049 | 4.62088 | 4.551252 | 0.691844 | 0.445579 | 1.335268 | 0.368684 | 0.656452 |
| E12 | RAD1 | 8.093507 | 8.477988 | 9.551491 | 7.287566 | 7.951983 | 1.067903 | 0.111914 | 1.167277 | 1.007509 | 1.163459 |
| F01 | RAD17 | 6.965633 | 8.207185 | 7.807465 | 6.145194 | 6.863013 | 1.305759 | 0.608902 | 1.618413 | 1.045261 | 1.233277 |
| F02 | RAD18 | 7.007127 | 8.89671 | 8.330852 | 6.556246 | 7.095146 | 0.829744 | 0.736697 | 1.299312 | 0.614477 | 1.00748 |
| F03 | RAD21 | 4.074715 | 5.872593 | 5.232791 | 3.800684 | 4.206946 | 0.978105 | 0.604481 | 0.911481 | 0.747966 | 1.076875 |
| F04 | RAD50 | 9.800364 | 11.062052 | 10.524121 | 10.308072 | 10.604617 | 0.927505 | 0.437774 | 1.45493 | 1.154851 | 2.474656 |
| F05 | RAD51 | 12.205949 | 14.076135 | 13.690672 | 11.038922 | 11.582845 | 0.893006 | 0.492845 | 1.089526 | 0.824176 | 1.078709 |
| F06 | RAD51L1 | 8.207763 | 9.737355 | 9.134756 | 8.024424 | 8.228559 | 1.067323 | 0.412877 | 1.295833 | 0.245412 | 1.142576 |
| F07 | RAD9A | 9.844277 | 11.416071 | 11.267483 | 9.368675 | 9.692969 | 1.484733 | 0.350282 | 1.424726 | 0.799504 | 0.679805 |
| F08 | RBBP8 | 6.23206 | 8.210676 | 7.656347 | 5.302078 | 6.166431 | 1.106489 | 0.726395 | 1.710802 | 1.130486 | 1.239072 |
| F09 | REV1 | 8.020581 | 8.988958 | 9.143459 | 7.38976 | 8.044459 | 1.023048 | 0.247055 | 1.55534 | 0.603595 | 1.184364 |
| F10 | RPA1 | 5.813524 | 7.582175 | 7.378143 | 5.816447 | 5.661629 | 0.79976 | 0.393952 | 1.009714 | 0.210619 | 0.825065 |
| F11 | SEMA4A | 12.502769 | 14.787919 | 16.931231 | 12.2366 | 12.767819 | 1.205837 | 0.761597 | 1.30702 | 1.429689 | 1.032686 |
| F12 | SESN1 | 7.756419 | 9.907502 | 9.007638 | 7.671683 | 7.902929 | 1.453499 | 0.89356 | 1.117511 | 0.983868 | 1.337775 |
| G01 | SMC1A | 5.894722 | 7.681258 | 7.947324 | 5.354623 | 6.048758 | 0.88664 | 0.791809 | 1.366268 | 0.353497 | 0.760151 |
| G02 | SUMO1 | 5.188013 | 6.526687 | 6.259738 | 4.510534 | 5.210804 | 0.813326 | 0.66683 | 1.176217 | 1.19366 | 1.118975 |
| G03 | TP53 | 5.05339 | 6.956738 | 6.303352 | 4.453644 | 4.905152 | 0.783164 | 0.790779 | 0.874968 | 0.953021 | 0.748727 |
| G04 | TP73 | 15.575853 | 18.554919 | 18.612993 | 16.055046 | 16.718136 | 2.087983 | 0.292848 | 0.966319 | 1.194976 | 1.793449 |
| G05 | TREX1 | 11.045072 | 10.014703 | 9.173274 | 10.379534 | 11.051392 | 1.271858 | 0.620273 | 1.369366 | 0.948466 | 1.251993 |
| G06 | UNG | 5.211392 | 8.778849 | 8.079468 | 4.018634 | 4.817068 | 0.880544 | 0.424857 | 1.271775 | 1.280883 | 0.901817 |
| G07 | XPA | 8.720267 | 8.465048 | 8.6261 | 8.240182 | 8.602831 | 1.312624 | 0.372525 | 1.287332 | 0.897557 | 1.276047 |
| G08 | XPC | 7.601959 | 8.115531 | 7.661842 | 6.716466 | 7.436431 | 1.269831 | 0.345479 | 1.127478 | 0.755709 | 1.320205 |
| G09 | XRCC1 | 8.216757 | 9.483348 | 9.241377 | 7.524547 | 8.175009 | 1.035185 | 0.340655 | 1.076963 | 0.661929 | 0.71196 |
| G10 | XRCC2 | 7.65112 | 11.859995 | 11.069672 | 7.016484 | 7.521846 | 0.688584 | 1.237798 | 1.67781 | 0.505821 | 1.151688 |
| G11 | XRCC3 | 12.194456 | 12.379706 | 13.386506 | 12.625713 | 12.643043 | 1.369984 | 0.688841 | 1.805186 | 1.03403 | 0.953933 |
| G12 | ZAK | 9.356967 | 6.096892 | 7.406611 | 9.091263 | 9.103565 | 1.067959 | 0.095853 | 1.186833 | 0.364457 | 1.178815 |
| H01 | B2M | 5.809935 | 2.649844 | 2.364349 | 5.439099 | 5.79029 | 1.33872 | 0.518214 | 1.162014 | 0.892604 | 1.101036 |
| H02 | HPRT1 | 4.890593 | 7.141134 | 5.729746 | 4.770403 | 5.358898 | 0.893559 | 0.751737 | 1.283548 | 1.122995 | 0.992357 |
| H03 | RPL13A | 2.025029 | 2.839781 | 2.552378 | 1.498381 | 2.361105 | 1.089475 | 0.253005 | 1.112723 | 0.971366 | 1.16708 |
| H04 | GAPDH | 0.723658 | 0.20212 | 0.998511 | -0.304185 | 0.658135 | 1.194777 | 0.810668 | 1.389459 | 1.584808 | 1.073441 |
| H05 | ACTB | 0 | 0 | 0 | 0 | 0 | 0 | 0 | 0 | 0 | 0 |
| H06 | HGDC | 15.484358 | 15.700479 | 15.401467 | 16.729387 | 15.145245 | 1.777959 | 1.868002 | 1.848803 | 1.021192 | 1.774062 |
| H07 | RTC | 6.036955 | 5.953932 | 5.994655 | 5.055941 | 5.770591 | 1.316973 | 0.776011 | 0.907468 | 0.627479 | 1.062766 |
| H08 | RTC | 6.02574 | 5.914898 | 6.004925 | 4.87783 | 5.809098 | 1.295676 | 0.752694 | 0.941241 | 0.784928 | 1.01361 |
| H09 | RTC | 6.043304 | 5.956343 | 6.035493 | 4.958104 | 5.909297 | 1.316573 | 0.719831 | 0.855408 | 0.705358 | 0.992175 |
| H10 | PPC | 2.881299 | 2.845937 | 2.962152 | 2.42013 | 2.630613 | 0.934 | 0.301844 | 0.831607 | 0.900318 | 0.91994 |
| H11 | PPC | 2.953596 | 2.730721 | 3.057955 | 2.555946 | 2.808222 | 1.013764 | 0.305188 | 0.825248 | 0.656645 | 0.969543 |
| H12 | PPC | 2.962801 | 2.859731 | 2.938244 | 2.092996 | 2.768027 | 1.036894 | 0.2804 | 0.853946 | 1.362636 | 0.925055 |

**D:** 2^(-Avg.(Delta(Ct))

| **Well** | **Symbol** | **IMR-90 iPS** | **IMR-90** | **TF** | **AE iPS** | **Control Group (ES)** |
| --- | --- | --- | --- | --- | --- | --- |
| A01 | ABL1 | 0.0065 | 0.009362 | 0.004019 | 0.008925 | 0.006619 |
| A02 | ANKRD17 | 0.012355 | 0.006488 | 0.005906 | 0.014472 | 0.011622 |
| A03 | APEX1 | 0.040917 | 0.009657 | 0.020774 | 0.087722 | 0.04574 |
| A04 | ATM | 0.012332 | 0.00334 | 0.005638 | 0.003979 | 0.008702 |
| A05 | ATR | 0.014366 | 0.002914 | 0.005705 | 0.007195 | 0.011754 |
| A06 | ATRX | 0.000959 | 0.000513 | 0.000291 | 0.001521 | 0.001166 |
| A07 | BRCA1 | 0.003011 | 0.000296 | 0.000716 | 0.003448 | 0.002975 |
| A08 | BTG2 | 0.000155 | 0.000126 | 0.000196 | 0.000062 | 0.000136 |
| A09 | CCNH | 0.010417 | 0.006287 | 0.009645 | 0.016675 | 0.009958 |
| A10 | CDK7 | 0.01524 | 0.017616 | 0.014884 | 0.026582 | 0.015488 |
| A11 | CHEK1 | 0.02162 | 0.001955 | 0.003736 | 0.04385 | 0.020863 |
| A12 | CHEK2 | 0.005002 | 0.000502 | 0.000617 | 0.006781 | 0.005822 |
| B01 | CIB1 | 0.006763 | 0.015436 | 0.020105 | 0.012984 | 0.007686 |
| B02 | CIDEA | 0.000003 | 0.000007 | 0.00003 | 0.000008 | 0.000006 |
| B03 | CRY1 | 0.008094 | 0.004375 | 0.003532 | 0.011001 | 0.008193 |
| B04 | DDB1 | 0.019623 | 0.013819 | 0.010553 | 0.021016 | 0.017489 |
| B05 | DDIT3 | 0.006043 | 0.00523 | 0.011696 | 0.004008 | 0.001786 |
| B06 | DMC1 | 0.000267 | 0.000006 | 0.000057 | 0.000399 | 0.000402 |
| B07 | ERCC1 | 0.002373 | 0.002935 | 0.003393 | 0.002584 | 0.002152 |
| B08 | ERCC2 | 0.005717 | 0.002329 | 0.002457 | 0.005451 | 0.004401 |
| B09 | EXO1 | 0.004609 | 0.000503 | 0.000897 | 0.009056 | 0.005949 |
| B10 | FANCG | 0.003862 | 0.000687 | 0.000921 | 0.004859 | 0.004019 |
| B11 | FEN1 | 0.011623 | 0.001358 | 0.002393 | 0.012002 | 0.009817 |
| B12 | XRCC6 | 0.021226 | 0.005392 | 0.007469 | 0.026929 | 0.022311 |
| C01 | GADD45A | 0.000994 | 0.00132 | 0.002172 | 0.00079 | 0.000782 |
| C02 | GADD45G | 0.001057 | 0.000038 | 0.000044 | 0.000583 | 0.000345 |
| C03 | GML | 0.000004 | 0.000003 | 0.000002 | 0.000004 | 0.000004 |
| C04 | GTF2H1 | 0.00457 | 0.00485 | 0.010851 | 0.009108 | 0.004955 |
| C05 | GTF2H2 | 0.017531 | 0.005095 | 0.005177 | 0.015234 | 0.013206 |
| C06 | GTSE1 | 0.002478 | 0.00031 | 0.000472 | 0.004751 | 0.00305 |
| C07 | HUS1 | 0.00186 | 0.000635 | 0.001053 | 0.002198 | 0.002052 |
| C08 | IGHMBP2 | 0.001886 | 0.001064 | 0.000527 | 0.001705 | 0.001181 |
| C09 | IP6K3 | 0.000041 | 0.000024 | 0.000039 | 0.000044 | 0.000049 |
| C10 | XRCC6BP1 | 0.000779 | 0.000244 | 0.000559 | 0.001505 | 0.000998 |
| C11 | LIG1 | 0.002333 | 0.000269 | 0.000478 | 0.003032 | 0.002419 |
| C12 | MAP2K6 | 0.004028 | 0.000308 | 0.001061 | 0.010684 | 0.007531 |
| D01 | MAPK12 | 0.002095 | 0.00397 | 0.003395 | 0.001699 | 0.001716 |
| D02 | MBD4 | 0.012473 | 0.009629 | 0.006252 | 0.021416 | 0.013362 |
| D03 | MLH1 | 0.012768 | 0.005149 | 0.007194 | 0.028732 | 0.018127 |
| D04 | MLH3 | 0.002299 | 0.000917 | 0.000785 | 0.002616 | 0.001884 |
| D05 | MNAT1 | 0.009449 | 0.006173 | 0.007187 | 0.01478 | 0.009325 |
| D06 | MPG | 0.006747 | 0.014055 | 0.013705 | 0.007777 | 0.006493 |
| D07 | MRE11A | 0.001058 | 0.000386 | 0.000335 | 0.00135 | 0.00157 |
| D08 | MSH2 | 0.063842 | 0.002702 | 0.005445 | 0.097472 | 0.058837 |
| D09 | MSH3 | 0.003852 | 0.001859 | 0.002591 | 0.005282 | 0.003683 |
| D10 | MUTYH | 0.004251 | 0.00047 | 0.000594 | 0.005113 | 0.0033 |
| D11 | N4BP2 | 0.006275 | 0.000627 | 0.000959 | 0.008209 | 0.006093 |
| D12 | NBN | 0.001486 | 0.000451 | 0.001052 | 0.001628 | 0.001596 |
| E01 | NTHL1 | 0.007652 | 0.001601 | 0.00184 | 0.013946 | 0.006861 |
| E02 | OGG1 | 0.004964 | 0.002551 | 0.003457 | 0.006851 | 0.005185 |
| E03 | PCBP4 | 0.000053 | 0.000105 | 0.000084 | 0.00002 | 0.000028 |
| E04 | PCNA | 0.072228 | 0.013122 | 0.021874 | 0.12287 | 0.080696 |
| E05 | AIFM1 | 0.011522 | 0.00174 | 0.005257 | 0.008087 | 0.00738 |
| E06 | PMS1 | 0.014275 | 0.002841 | 0.003776 | 0.018406 | 0.011936 |
| E07 | PMS2 | 0.004614 | 0.003181 | 0.002619 | 0.0048 | 0.003146 |
| E08 | PMS2L3 | 0.009293 | 0.009119 | 0.004687 | 0.010442 | 0.008344 |
| E09 | PNKP | 0.004595 | 0.001844 | 0.001959 | 0.004465 | 0.003224 |
| E10 | PPP1R15A | 0.000993 | 0.003173 | 0.003134 | 0.000493 | 0.000665 |
| E11 | PRKDC | 0.03994 | 0.008192 | 0.010166 | 0.040642 | 0.042652 |
| E12 | RAD1 | 0.003661 | 0.002805 | 0.001333 | 0.006401 | 0.004038 |
| F01 | RAD17 | 0.008001 | 0.003384 | 0.004464 | 0.014129 | 0.008591 |
| F02 | RAD18 | 0.007774 | 0.002098 | 0.003106 | 0.010626 | 0.007314 |
| F03 | RAD21 | 0.059346 | 0.017068 | 0.026593 | 0.07176 | 0.054148 |
| F04 | RAD50 | 0.001121 | 0.000468 | 0.000679 | 0.000789 | 0.000642 |
| F05 | RAD51 | 0.000212 | 0.000058 | 0.000076 | 0.000475 | 0.000326 |
| F06 | RAD51L1 | 0.003382 | 0.001172 | 0.001779 | 0.003841 | 0.003334 |
| F07 | RAD9A | 0.001088 | 0.000366 | 0.000406 | 0.001513 | 0.001208 |
| F08 | RBBP8 | 0.013303 | 0.003376 | 0.004957 | 0.025346 | 0.013923 |
| F09 | REV1 | 0.003851 | 0.001968 | 0.001768 | 0.005963 | 0.003788 |
| F10 | RPA1 | 0.017781 | 0.005218 | 0.006011 | 0.017745 | 0.019755 |
| F11 | SEMA4A | 0.000172 | 0.000035 | 0.000008 | 0.000207 | 0.000143 |
| F12 | SESN1 | 0.004625 | 0.001041 | 0.001943 | 0.004904 | 0.004178 |
| G01 | SMC1A | 0.016808 | 0.004872 | 0.004052 | 0.02444 | 0.015106 |
| G02 | SUMO1 | 0.027432 | 0.010846 | 0.013051 | 0.043873 | 0.027002 |
| G03 | TP53 | 0.030115 | 0.00805 | 0.012662 | 0.045637 | 0.033374 |
| G04 | TP73 | 0.00002 | 0.000003 | 0.000002 | 0.000015 | 0.000009 |
| G05 | TREX1 | 0.000473 | 0.000967 | 0.001732 | 0.000751 | 0.000471 |
| G06 | UNG | 0.026991 | 0.002277 | 0.003697 | 0.061698 | 0.035475 |
| G07 | XPA | 0.002371 | 0.00283 | 0.002531 | 0.003307 | 0.002572 |
| G08 | XPC | 0.005147 | 0.003606 | 0.004938 | 0.009509 | 0.005773 |
| G09 | XRCC1 | 0.003361 | 0.001397 | 0.001652 | 0.005431 | 0.00346 |
| G10 | XRCC2 | 0.004975 | 0.000269 | 0.000465 | 0.007724 | 0.005441 |
| G11 | XRCC3 | 0.000213 | 0.000188 | 0.000093 | 0.000158 | 0.000156 |
| G12 | ZAK | 0.001525 | 0.01461 | 0.005894 | 0.001833 | 0.001818 |
| H01 | B2M | 0.017825 | 0.159337 | 0.194205 | 0.02305 | 0.01807 |
| H02 | HPRT1 | 0.033712 | 0.007084 | 0.018844 | 0.036641 | 0.024367 |
| H03 | RPL13A | 0.2457 | 0.139682 | 0.170474 | 0.35395 | 0.194642 |
| H04 | GAPDH | 0.60556 | 0.869272 | 0.500516 | 1.234721 | 0.633697 |
| H05 | ACTB | 1 | 1 | 1 | 1 | 1 |
| H06 | HGDC | 0.000022 | 0.000019 | 0.000023 | 0.000009 | 0.000028 |
| H07 | RTC | 0.01523 | 0.016132 | 0.015683 | 0.030061 | 0.018318 |
| H08 | RTC | 0.015349 | 0.016574 | 0.015572 | 0.034012 | 0.017836 |
| H09 | RTC | 0.015163 | 0.016105 | 0.015245 | 0.032171 | 0.016639 |
| H10 | PPC | 0.13572 | 0.139087 | 0.128323 | 0.186839 | 0.161475 |
| H11 | PPC | 0.129086 | 0.150651 | 0.120078 | 0.170053 | 0.142771 |
| H12 | PPC | 0.128265 | 0.137764 | 0.130467 | 0.234393 | 0.146805 |

**E: Fold Change (comparing to ES cells) and 95% confidence intervals.**

| **Well** | **Symbol** | **IMR-90 iPS** | **95% CI** | **IMR-90** | **95% CI** | **TF** | **95% CI** | **AE iPS** | **95% CI** |
| --- | --- | --- | --- | --- | --- | --- | --- | --- | --- |
| A01 | ABL1 | 0.9819 | ( 0.30, 1.66 ) | 1.4143 | ( 0.79, 2.04 ) | 0.6072 | ( 0.00001, 1.31 ) | 1.3484 | ( 0.38, 2.32 ) |
| A02 | ANKRD17 | 1.063 | ( 0.46, 1.67 ) | 0.5582 | ( 0.32, 0.79 ) | 0.5082 | ( 0.00001, 1.02 ) | 1.2452 | ( 0.73, 1.76 ) |
| A03 | APEX1 | 0.8946 | ( 0.00001, 1.81 ) | 0.2111 | ( 0.02, 0.40 ) | 0.4542 | ( 0.00001, 1.10 ) | 1.9179 | ( 0.00001, 4.31 ) |
| A04 | ATM | 1.417 | ( 0.00001, 3.17 ) | 0.3838 | ( 0.00001, 0.78 ) | 0.6479 | ( 0.00001, 1.40 ) | 0.4572 | ( 0.00001, 1.30 ) |
| A05 | ATR | 1.2223 | ( 0.92, 1.53 ) | 0.2479 | ( 0.16, 0.33 ) | 0.4854 | ( 0.29, 0.68 ) | 0.6121 | ( 0.15, 1.07 ) |
| A06 | ATRX | 0.8218 | ( 0.08, 1.57 ) | 0.44 | ( 0.18, 0.70 ) | 0.2494 | ( 0.00001, 0.58 ) | 1.3041 | ( 0.42, 2.18 ) |
| A07 | BRCA1 | 1.012 | ( 0.32, 1.70 ) | 0.0995 | ( 0.02, 0.18 ) | 0.2407 | ( 0.00001, 0.60 ) | 1.1589 | ( 0.52, 1.80 ) |
| A08 | BTG2 | 1.1339 | ( 0.00001, 3.11 ) | 0.9264 | ( 0.11, 1.75 ) | 1.4385 | ( 0.00001, 3.05 ) | 0.4542 | ( 0.12, 0.79 ) |
| A09 | CCNH | 1.0461 | ( 0.06, 2.03 ) | 0.6314 | ( 0.25, 1.02 ) | 0.9686 | ( 0.00001, 1.97 ) | 1.6746 | ( 0.16, 3.19 ) |
| A10 | CDK7 | 0.984 | ( 0.17, 1.79 ) | 1.1374 | ( 0.58, 1.69 ) | 0.961 | ( 0.29, 1.64 ) | 1.7163 | ( 0.17, 3.26 ) |
| A11 | CHEK1 | 1.0363 | ( 0.18, 1.89 ) | 0.0937 | ( 0.02, 0.17 ) | 0.179 | ( 0.00001, 0.42 ) | 2.1018 | ( 0.00001, 4.40 ) |
| A12 | CHEK2 | 0.8592 | ( 0.01, 1.71 ) | 0.0862 | ( 0.03, 0.14 ) | 0.106 | ( 0.00001, 0.23 ) | 1.1648 | ( 0.46, 1.87 ) |
| B01 | CIB1 | 0.88 | ( 0.00001, 1.77 ) | 2.0084 | ( 0.74, 3.28 ) | 2.6159 | ( 0.00001, 5.99 ) | 1.6894 | ( 0.00001, 3.68 ) |
| B02 | CIDEA | 0.5314 | ( 0.00001, 1.32 ) | 1.0626 | ( 0.00001, 2.52 ) | 4.5681 | ( 0.00001, 10.30 ) | 1.2276 | ( 0.00001, 2.53 ) |
| B03 | CRY1 | 0.988 | ( 0.05, 1.93 ) | 0.534 | ( 0.20, 0.86 ) | 0.4312 | ( 0.07, 0.79 ) | 1.3428 | ( 0.24, 2.44 ) |
| B04 | DDB1 | 1.122 | ( 0.45, 1.79 ) | 0.7902 | ( 0.45, 1.13 ) | 0.6034 | ( 0.15, 1.06 ) | 1.2017 | ( 0.46, 1.94 ) |
| B05 | DDIT3 | 3.3842 | ( 0.00001, 8.27 ) | 2.929 | ( 0.00001, 5.95 ) | 6.5497 | ( 0.00001, 15.90 ) | 2.2442 | ( 0.00001, 5.55 ) |
| B06 | DMC1 | 0.6628 | ( 0.08, 1.25 ) | 0.0156 | ( 0.00, 0.03 ) | 0.1422 | ( 0.00001, 0.38 ) | 0.9927 | ( 0.00001, 2.05 ) |
| B07 | ERCC1 | 1.1026 | ( 0.00001, 2.38 ) | 1.3641 | ( 0.47, 2.26 ) | 1.5768 | ( 0.00001, 3.35 ) | 1.201 | ( 0.00001, 2.61 ) |
| B08 | ERCC2 | 1.2989 | ( 0.22, 2.38 ) | 0.5291 | ( 0.27, 0.79 ) | 0.5582 | ( 0.06, 1.06 ) | 1.2386 | ( 0.46, 2.01 ) |
| B09 | EXO1 | 0.7748 | ( 0.22, 1.33 ) | 0.0846 | ( 0.00001, 0.17 ) | 0.1509 | ( 0.00001, 0.37 ) | 1.5222 | ( 0.45, 2.59 ) |
| B10 | FANCG | 0.9609 | ( 0.42, 1.50 ) | 0.1708 | ( 0.09, 0.25 ) | 0.2291 | ( 0.00001, 0.50 ) | 1.2089 | ( 0.43, 1.98 ) |
| B11 | FEN1 | 1.1839 | ( 0.54, 1.83 ) | 0.1384 | ( 0.04, 0.24 ) | 0.2438 | ( 0.00001, 0.52 ) | 1.2226 | ( 0.24, 2.21 ) |
| B12 | XRCC6 | 0.9514 | ( 0.22, 1.69 ) | 0.2417 | ( 0.12, 0.36 ) | 0.3348 | ( 0.00001, 0.67 ) | 1.207 | ( 0.04, 2.37 ) |
| C01 | GADD45A | 1.2718 | ( 0.00001, 2.77 ) | 1.6887 | ( 0.07, 3.30 ) | 2.7789 | ( 0.00001, 5.88 ) | 1.0103 | ( 0.00001, 2.19 ) |
| C02 | GADD45G | 3.0666 | ( 0.00001, 8.17 ) | 0.1101 | ( 0.01, 0.21 ) | 0.1279 | ( 0.00001, 0.28 ) | 1.6917 | ( 0.18, 3.21 ) |
| C03 | GML | 0.8744 | ( 0.00001, 2.12 ) | 0.5792 | ( 0.00001, 1.24 ) | 0.5563 | ( 0.00001, 1.31 ) | 0.9549 | ( 0.00001, 2.34 ) |
| C04 | GTF2H1 | 0.9223 | ( 0.14, 1.70 ) | 0.9787 | ( 0.30, 1.65 ) | 2.1899 | ( 0.00001, 4.79 ) | 1.8381 | ( 0.00001, 4.00 ) |
| C05 | GTF2H2 | 1.3275 | ( 0.42, 2.24 ) | 0.3858 | ( 0.18, 0.59 ) | 0.392 | ( 0.00001, 0.82 ) | 1.1536 | ( 0.19, 2.12 ) |
| C06 | GTSE1 | 0.8125 | ( 0.08, 1.55 ) | 0.1018 | ( 0.02, 0.19 ) | 0.1549 | ( 0.00001, 0.52 ) | 1.5579 | ( 0.00001, 3.56 ) |
| C07 | HUS1 | 0.9065 | ( 0.21, 1.61 ) | 0.3095 | ( 0.02, 0.60 ) | 0.513 | ( 0.00001, 1.06 ) | 1.0711 | ( 0.32, 1.82 ) |
| C08 | IGHMBP2 | 1.5974 | ( 0.34, 2.86 ) | 0.9014 | ( 0.57, 1.24 ) | 0.4459 | ( 0.05, 0.84 ) | 1.4436 | ( 0.65, 2.24 ) |
| C09 | IP6K3 | 0.8376 | ( 0.00001, 2.07 ) | 0.4849 | ( 0.00001, 1.17 ) | 0.7855 | ( 0.00001, 2.13 ) | 0.8905 | ( 0.00001, 1.79 ) |
| C10 | XRCC6BP1 | 0.7806 | ( 0.00001, 1.59 ) | 0.2444 | ( 0.11, 0.38 ) | 0.5604 | ( 0.00001, 1.20 ) | 1.5083 | ( 0.00001, 3.14 ) |
| C11 | LIG1 | 0.9646 | ( 0.26, 1.67 ) | 0.1113 | ( 0.04, 0.19 ) | 0.1977 | ( 0.00001, 0.45 ) | 1.2533 | ( 0.30, 2.21 ) |
| C12 | MAP2K6 | 0.5349 | ( 0.26, 0.81 ) | 0.0409 | ( 0.02, 0.06 ) | 0.1408 | ( 0.05, 0.24 ) | 1.4187 | ( 0.10, 2.74 ) |
| D01 | MAPK12 | 1.2207 | ( 0.15, 2.29 ) | 2.3137 | ( 1.32, 3.31 ) | 1.9787 | ( 0.04, 3.92 ) | 0.9898 | ( 0.44, 1.54 ) |
| D02 | MBD4 | 0.9335 | ( 0.16, 1.71 ) | 0.7207 | ( 0.36, 1.08 ) | 0.4679 | ( 0.01, 0.92 ) | 1.6028 | ( 0.20, 3.00 ) |
| D03 | MLH1 | 0.7043 | ( 0.14, 1.27 ) | 0.284 | ( 0.10, 0.47 ) | 0.3969 | ( 0.00001, 0.85 ) | 1.585 | ( 0.05, 3.12 ) |
| D04 | MLH3 | 1.2202 | ( 0.18, 2.26 ) | 0.4866 | ( 0.19, 0.79 ) | 0.4168 | ( 0.00001, 1.05 ) | 1.3887 | ( 0.48, 2.29 ) |
| D05 | MNAT1 | 1.0133 | ( 0.04, 1.99 ) | 0.662 | ( 0.21, 1.11 ) | 0.7708 | ( 0.00001, 1.62 ) | 1.585 | ( 0.11, 3.06 ) |
| D06 | MPG | 1.0391 | ( 0.00001, 2.09 ) | 2.1646 | ( 1.27, 3.06 ) | 2.1108 | ( 0.00001, 4.35 ) | 1.1978 | ( 0.00001, 2.47 ) |
| D07 | MRE11A | 0.6737 | ( 0.16, 1.19 ) | 0.2459 | ( 0.14, 0.35 ) | 0.2131 | ( 0.00001, 0.43 ) | 0.8596 | ( 0.42, 1.30 ) |
| D08 | MSH2 | 1.0851 | ( 0.09, 2.08 ) | 0.0459 | ( 0.02, 0.08 ) | 0.0926 | ( 0.00001, 0.20 ) | 1.6566 | ( 0.40, 2.92 ) |
| D09 | MSH3 | 1.046 | ( 0.47, 1.62 ) | 0.5046 | ( 0.34, 0.67 ) | 0.7034 | ( 0.10, 1.31 ) | 1.4342 | ( 0.82, 2.05 ) |
| D10 | MUTYH | 1.2883 | ( 0.37, 2.20 ) | 0.1423 | ( 0.07, 0.21 ) | 0.1799 | ( 0.00001, 0.38 ) | 1.5494 | ( 0.39, 2.70 ) |
| D11 | N4BP2 | 1.0298 | ( 0.39, 1.67 ) | 0.1029 | ( 0.04, 0.16 ) | 0.1574 | ( 0.00001, 0.33 ) | 1.3472 | ( 0.62, 2.07 ) |
| D12 | NBN | 0.9314 | ( 0.10, 1.76 ) | 0.2826 | ( 0.12, 0.45 ) | 0.6591 | ( 0.00001, 1.40 ) | 1.0203 | ( 0.48, 1.56 ) |
| E01 | NTHL1 | 1.1154 | ( 0.10, 2.13 ) | 0.2333 | ( 0.10, 0.36 ) | 0.2682 | ( 0.00001, 0.63 ) | 2.0327 | ( 0.00001, 4.36 ) |
| E02 | OGG1 | 0.9575 | ( 0.28, 1.63 ) | 0.4919 | ( 0.24, 0.75 ) | 0.6667 | ( 0.00001, 1.36 ) | 1.3214 | ( 0.09, 2.56 ) |
| E03 | PCBP4 | 1.9099 | ( 0.27, 3.55 ) | 3.7793 | ( 0.49, 7.07 ) | 3.0362 | ( 0.00001, 6.13 ) | 0.7031 | ( 0.00001, 1.44 ) |
| E04 | PCNA | 0.8951 | ( 0.22, 1.57 ) | 0.1626 | ( 0.05, 0.27 ) | 0.2711 | ( 0.00001, 0.61 ) | 1.5226 | ( 0.00001, 3.17 ) |
| E05 | AIFM1 | 1.5613 | ( 0.23, 2.90 ) | 0.2358 | ( 0.09, 0.38 ) | 0.7124 | ( 0.00001, 1.46 ) | 1.0959 | ( 0.05, 2.14 ) |
| E06 | PMS1 | 1.196 | ( 0.20, 2.19 ) | 0.238 | ( 0.11, 0.37 ) | 0.3164 | ( 0.00001, 0.66 ) | 1.5421 | ( 0.48, 2.60 ) |
| E07 | PMS2 | 1.4665 | ( 0.17, 2.76 ) | 1.0111 | ( 0.56, 1.46 ) | 0.8324 | ( 0.00001, 1.76 ) | 1.5256 | ( 0.65, 2.40 ) |
| E08 | PMS2L3 | 1.1137 | ( 0.21, 2.01 ) | 1.0928 | ( 0.58, 1.61 ) | 0.5618 | ( 0.00001, 1.32 ) | 1.2514 | ( 0.61, 1.89 ) |
| E09 | PNKP | 1.4253 | ( 0.03, 2.82 ) | 0.5722 | ( 0.28, 0.87 ) | 0.6078 | ( 0.00001, 1.36 ) | 1.3852 | ( 0.03, 2.74 ) |
| E10 | PPP1R15A | 1.4918 | ( 0.00001, 3.58 ) | 4.7687 | ( 2.89, 6.64 ) | 4.7095 | ( 0.00001, 9.91 ) | 0.7413 | ( 0.14, 1.34 ) |
| E11 | PRKDC | 0.9364 | ( 0.42, 1.45 ) | 0.1921 | ( 0.11, 0.27 ) | 0.2384 | ( 0.00001, 0.50 ) | 0.9529 | ( 0.57, 1.34 ) |
| E12 | RAD1 | 0.9066 | ( 0.11, 1.71 ) | 0.6945 | ( 0.34, 1.05 ) | 0.33 | ( 0.00001, 0.67 ) | 1.5849 | ( 0.10, 3.07 ) |
| F01 | RAD17 | 0.9313 | ( 0.00001, 1.89 ) | 0.3939 | ( 0.13, 0.66 ) | 0.5196 | ( 0.00001, 1.23 ) | 1.6447 | ( 0.04, 3.25 ) |
| F02 | RAD18 | 1.0629 | ( 0.31, 1.82 ) | 0.2869 | ( 0.10, 0.48 ) | 0.4246 | ( 0.00001, 0.89 ) | 1.4529 | ( 0.51, 2.39 ) |
| F03 | RAD21 | 1.096 | ( 0.21, 1.98 ) | 0.3152 | ( 0.12, 0.51 ) | 0.4911 | ( 0.07, 0.91 ) | 1.3252 | ( 0.34, 2.32 ) |
| F04 | RAD50 | 1.7462 | ( 0.00001, 3.90 ) | 0.7283 | ( 0.00001, 1.53 ) | 1.0574 | ( 0.00001, 2.71 ) | 1.2282 | ( 0.00001, 2.94 ) |
| F05 | RAD51 | 0.6493 | ( 0.15, 1.14 ) | 0.1776 | ( 0.08, 0.28 ) | 0.232 | ( 0.01, 0.46 ) | 1.4579 | ( 0.30, 2.62 ) |
| F06 | RAD51L1 | 1.0145 | ( 0.13, 1.90 ) | 0.3514 | ( 0.15, 0.55 ) | 0.5336 | ( 0.00001, 1.14 ) | 1.152 | ( 0.54, 1.76 ) |
| F07 | RAD9A | 0.9004 | ( 0.00001, 1.85 ) | 0.3029 | ( 0.19, 0.42 ) | 0.3358 | ( 0.00001, 0.72 ) | 1.2521 | ( 0.39, 2.12 ) |
| F08 | RBBP8 | 0.9555 | ( 0.08, 1.84 ) | 0.2424 | ( 0.07, 0.42 ) | 0.356 | ( 0.00001, 0.87 ) | 1.8205 | ( 0.00001, 3.70 ) |
| F09 | REV1 | 1.0167 | ( 0.14, 1.89 ) | 0.5196 | ( 0.24, 0.80 ) | 0.4668 | ( 0.00001, 1.08 ) | 1.5743 | ( 0.48, 2.67 ) |
| F10 | RPA1 | 0.9001 | ( 0.32, 1.48 ) | 0.2642 | ( 0.15, 0.38 ) | 0.3043 | ( 0.04, 0.57 ) | 0.8982 | ( 0.55, 1.25 ) |
| F11 | SEMA4A | 1.2017 | ( 0.08, 2.32 ) | 0.2465 | ( 0.08, 0.41 ) | 0.0558 | ( 0.00001, 0.12 ) | 1.4451 | ( 0.00001, 3.19 ) |
| F12 | SESN1 | 1.1069 | ( 0.00001, 2.37 ) | 0.2492 | ( 0.04, 0.46 ) | 0.465 | ( 0.00001, 0.95 ) | 1.1738 | ( 0.04, 2.30 ) |
| G01 | SMC1A | 1.1127 | ( 0.35, 1.87 ) | 0.3225 | ( 0.12, 0.53 ) | 0.2682 | ( 0.00001, 0.57 ) | 1.6179 | ( 0.92, 2.31 ) |
| G02 | SUMO1 | 1.0159 | ( 0.27, 1.76 ) | 0.4017 | ( 0.14, 0.67 ) | 0.4833 | ( 0.00001, 0.99 ) | 1.6248 | ( 0.00001, 3.33 ) |
| G03 | TP53 | 0.9024 | ( 0.34, 1.46 ) | 0.2412 | ( 0.09, 0.39 ) | 0.3794 | ( 0.09, 0.67 ) | 1.3675 | ( 0.25, 2.48 ) |
| G04 | TP73 | 2.2073 | ( 0.00001, 5.77 ) | 0.2799 | ( 0.06, 0.50 ) | 0.2689 | ( 0.00001, 0.56 ) | 1.5835 | ( 0.00001, 3.50 ) |
| G05 | TREX1 | 1.0044 | ( 0.00001, 2.03 ) | 2.0515 | ( 0.65, 3.45 ) | 3.676 | ( 0.00001, 8.09 ) | 1.5931 | ( 0.13, 3.06 ) |
| G06 | UNG | 0.7608 | ( 0.22, 1.30 ) | 0.0642 | ( 0.03, 0.10 ) | 0.1042 | ( 0.00001, 0.22 ) | 1.7392 | ( 0.00001, 3.61 ) |
| G07 | XPA | 0.9218 | ( 0.00001, 1.89 ) | 1.1002 | ( 0.44, 1.76 ) | 0.984 | ( 0.00001, 2.11 ) | 1.2858 | ( 0.14, 2.43 ) |
| G08 | XPC | 0.8916 | ( 0.00001, 1.81 ) | 0.6246 | ( 0.24, 1.01 ) | 0.8554 | ( 0.00001, 1.75 ) | 1.6471 | ( 0.30, 3.00 ) |
| G09 | XRCC1 | 0.9715 | ( 0.23, 1.72 ) | 0.4038 | ( 0.25, 0.56 ) | 0.4775 | ( 0.05, 0.91 ) | 1.5697 | ( 0.62, 2.52 ) |
| G10 | XRCC2 | 0.9143 | ( 0.29, 1.54 ) | 0.0494 | ( 0.00, 0.10 ) | 0.0855 | ( 0.00001, 0.21 ) | 1.4195 | ( 0.52, 2.32 ) |
| G11 | XRCC3 | 1.3647 | ( 0.00001, 2.75 ) | 1.2003 | ( 0.45, 1.95 ) | 0.5973 | ( 0.00001, 1.48 ) | 1.0121 | ( 0.09, 1.93 ) |
| G12 | ZAK | 0.8389 | ( 0.10, 1.58 ) | 8.0371 | ( 3.93, 12.14 ) | 3.2422 | ( 0.00001, 6.68 ) | 1.0086 | ( 0.42, 1.60 ) |
| H01 | B2M | 0.9865 | ( 0.00001, 2.00 ) | 8.818 | ( 3.62, 14.02 ) | 10.7476 | ( 0.00001, 21.78 ) | 1.2756 | ( 0.20, 2.35 ) |
| H02 | HPRT1 | 1.3835 | ( 0.36, 2.41 ) | 0.2907 | ( 0.10, 0.48 ) | 0.7733 | ( 0.00001, 1.62 ) | 1.5037 | ( 0.03, 2.98 ) |
| H03 | RPL13A | 1.2623 | ( 0.13, 2.39 ) | 0.7176 | ( 0.34, 1.10 ) | 0.8758 | ( 0.00001, 1.76 ) | 1.8185 | ( 0.16, 3.48 ) |
| H04 | GAPDH | 0.9556 | ( 0.06, 1.85 ) | 1.3717 | ( 0.39, 2.36 ) | 0.7898 | ( 0.00001, 1.72 ) | 1.9484 | ( 0.00001, 4.53 ) |
| H05 | ACTB | 1 | ( 1.00, 1.00 ) | 1 | ( 1.00, 1.00 ) | 1 | ( 1.00, 1.00 ) | 1 | ( 1.00, 1.00 ) |
| H06 | HGDC | 0.7905 | ( 0.00001, 1.92 ) | 0.6805 | ( 0.00001, 1.69 ) | 0.8373 | ( 0.00001, 2.21 ) | 0.3335 | ( 0.00001, 0.70 ) |
| H07 | RTC | 0.8314 | ( 0.00001, 1.67 ) | 0.8807 | ( 0.27, 1.49 ) | 0.8562 | ( 0.13, 1.58 ) | 1.6411 | ( 0.54, 2.74 ) |
| H08 | RTC | 0.8606 | ( 0.02, 1.71 ) | 0.9293 | ( 0.31, 1.55 ) | 0.8731 | ( 0.12, 1.62 ) | 1.907 | ( 0.47, 3.35 ) |
| H09 | RTC | 0.9113 | ( 0.01, 1.81 ) | 0.9679 | ( 0.34, 1.60 ) | 0.9162 | ( 0.19, 1.64 ) | 1.9335 | ( 0.58, 3.28 ) |
| H10 | PPC | 0.8405 | ( 0.21, 1.47 ) | 0.8614 | ( 0.48, 1.24 ) | 0.7947 | ( 0.19, 1.40 ) | 1.1571 | ( 0.22, 2.09 ) |
| H11 | PPC | 0.9041 | ( 0.18, 1.63 ) | 1.0552 | ( 0.56, 1.55 ) | 0.8411 | ( 0.19, 1.49 ) | 1.1911 | ( 0.40, 1.98 ) |
| H12 | PPC | 0.8737 | ( 0.17, 1.58 ) | 0.9384 | ( 0.52, 1.35 ) | 0.8887 | ( 0.20, 1.58 ) | 1.5966 | ( 0.00001, 3.42 ) |

**F: Fold regulation (up (+) or down (-) regulation relative to ES cells).**

| **Well** | **Symbol** | **IMR-90 iPS** | **IMR-90** | **TF** | **AE iPS** |
| --- | --- | --- | --- | --- | --- |
| A01 | ABL1 | -1.0184 | 1.4143 | -1.6469 | 1.3484 |
| A02 | ANKRD17 | 1.063 | -1.7913 | -1.9677 | 1.2452 |
| A03 | APEX1 | -1.1179 | -4.7364 | -2.2018 | 1.9179 |
| A04 | ATM | 1.417 | -2.6054 | -1.5435 | -2.1872 |
| A05 | ATR | 1.2223 | -4.0339 | -2.0603 | -1.6336 |
| A06 | ATRX | -1.2168 | -2.2727 | -4.0093 | 1.3041 |
| A07 | BRCA1 | 1.012 | -10.0467 | -4.1546 | 1.1589 |
| A08 | BTG2 | 1.1339 | -1.0794 | 1.4385 | -2.2015 |
| A09 | CCNH | 1.0461 | -1.5839 | -1.0324 | 1.6746 |
| A10 | CDK7 | -1.0163 | 1.1374 | -1.0406 | 1.7163 |
| A11 | CHEK1 | 1.0363 | -10.6724 | -5.5851 | 2.1018 |
| A12 | CHEK2 | -1.1639 | -11.5969 | -9.4351 | 1.1648 |
| B01 | CIB1 | -1.1364 | 2.0084 | 2.6159 | 1.6894 |
| B02 | CIDEA | -1.8819 | 1.0626 | 4.5681 | 1.2276 |
| B03 | CRY1 | -1.0122 | -1.8727 | -2.3194 | 1.3428 |
| B04 | DDB1 | 1.122 | -1.2655 | -1.6572 | 1.2017 |
| B05 | DDIT3 | 3.3842 | 2.929 | 6.5497 | 2.2442 |
| B06 | DMC1 | -1.5087 | -64.1249 | -7.0336 | -1.0073 |
| B07 | ERCC1 | 1.1026 | 1.3641 | 1.5768 | 1.201 |
| B08 | ERCC2 | 1.2989 | -1.8901 | -1.7916 | 1.2386 |
| B09 | EXO1 | -1.2906 | -11.8198 | -6.6289 | 1.5222 |
| B10 | FANCG | -1.0407 | -5.8538 | -4.3649 | 1.2089 |
| B11 | FEN1 | 1.1839 | -7.2269 | -4.1023 | 1.2226 |
| B12 | XRCC6 | -1.0511 | -4.1381 | -2.9871 | 1.207 |
| C01 | GADD45A | 1.2718 | 1.6887 | 2.7789 | 1.0103 |
| C02 | GADD45G | 3.0666 | -9.0795 | -7.8164 | 1.6917 |
| C03 | GML | -1.1436 | -1.7266 | -1.7975 | -1.0472 |
| C04 | GTF2H1 | -1.0843 | -1.0218 | 2.1899 | 1.8381 |
| C05 | GTF2H2 | 1.3275 | -2.5919 | -2.5508 | 1.1536 |
| C06 | GTSE1 | -1.2308 | -9.8235 | -6.4558 | 1.5579 |
| C07 | HUS1 | -1.1032 | -3.2305 | -1.9494 | 1.0711 |
| C08 | IGHMBP2 | 1.5974 | -1.1094 | -2.2426 | 1.4436 |
| C09 | IP6K3 | -1.1939 | -2.0622 | -1.2731 | -1.1229 |
| C10 | XRCC6BP1 | -1.2811 | -4.0922 | -1.7845 | 1.5083 |
| C11 | LIG1 | -1.0367 | -8.9831 | -5.058 | 1.2533 |
| C12 | MAP2K6 | -1.8696 | -24.4491 | -7.1001 | 1.4187 |
| D01 | MAPK12 | 1.2207 | 2.3137 | 1.9787 | -1.0103 |
| D02 | MBD4 | -1.0713 | -1.3876 | -2.1372 | 1.6028 |
| D03 | MLH1 | -1.4198 | -3.5205 | -2.5196 | 1.585 |
| D04 | MLH3 | 1.2202 | -2.0551 | -2.3994 | 1.3887 |
| D05 | MNAT1 | 1.0133 | -1.5107 | -1.2974 | 1.585 |
| D06 | MPG | 1.0391 | 2.1646 | 2.1108 | 1.1978 |
| D07 | MRE11A | -1.4844 | -4.0665 | -4.6931 | -1.1633 |
| D08 | MSH2 | 1.0851 | -21.7767 | -10.8048 | 1.6566 |
| D09 | MSH3 | 1.046 | -1.9817 | -1.4217 | 1.4342 |
| D10 | MUTYH | 1.2883 | -7.0281 | -5.5586 | 1.5494 |
| D11 | N4BP2 | 1.0298 | -9.7143 | -6.3524 | 1.3472 |
| D12 | NBN | -1.0737 | -3.5384 | -1.5172 | 1.0203 |
| E01 | NTHL1 | 1.1154 | -4.2865 | -3.7284 | 2.0327 |
| E02 | OGG1 | -1.0444 | -2.0328 | -1.4999 | 1.3214 |
| E03 | PCBP4 | 1.9099 | 3.7793 | 3.0362 | -1.4222 |
| E04 | PCNA | -1.1173 | -6.1499 | -3.6892 | 1.5226 |
| E05 | AIFM1 | 1.5613 | -4.2411 | -1.4038 | 1.0959 |
| E06 | PMS1 | 1.196 | -4.2009 | -3.161 | 1.5421 |
| E07 | PMS2 | 1.4665 | 1.0111 | -1.2013 | 1.5256 |
| E08 | PMS2L3 | 1.1137 | 1.0928 | -1.7801 | 1.2514 |
| E09 | PNKP | 1.4253 | -1.7477 | -1.6453 | 1.3852 |
| E10 | PPP1R15A | 1.4918 | 4.7687 | 4.7095 | -1.349 |
| E11 | PRKDC | -1.0679 | -5.2064 | -4.1954 | -1.0494 |
| E12 | RAD1 | -1.1031 | -1.4399 | -3.0304 | 1.5849 |
| F01 | RAD17 | -1.0737 | -2.5388 | -1.9245 | 1.6447 |
| F02 | RAD18 | 1.0629 | -3.486 | -2.355 | 1.4529 |
| F03 | RAD21 | 1.096 | -3.1726 | -2.0362 | 1.3252 |
| F04 | RAD50 | 1.7462 | -1.3731 | 1.0574 | 1.2282 |
| F05 | RAD51 | -1.5402 | -5.6306 | -4.3104 | 1.4579 |
| F06 | RAD51L1 | 1.0145 | -2.8457 | -1.8741 | 1.152 |
| F07 | RAD9A | -1.1106 | -3.3015 | -2.9784 | 1.2521 |
| F08 | RBBP8 | -1.0465 | -4.1246 | -2.8087 | 1.8205 |
| F09 | REV1 | 1.0167 | -1.9245 | -2.1421 | 1.5743 |
| F10 | RPA1 | -1.111 | -3.7857 | -3.2864 | -1.1133 |
| F11 | SEMA4A | 1.2017 | -4.0561 | -17.9189 | 1.4451 |
| F12 | SESN1 | 1.1069 | -4.0127 | -2.1506 | 1.1738 |
| G01 | SMC1A | 1.1127 | -3.1005 | -3.7284 | 1.6179 |
| G02 | SUMO1 | 1.0159 | -2.4895 | -2.069 | 1.6248 |
| G03 | TP53 | -1.1082 | -4.1456 | -2.6357 | 1.3675 |
| G04 | TP73 | 2.2073 | -3.5721 | -3.7189 | 1.5835 |
| G05 | TREX1 | 1.0044 | 2.0515 | 3.676 | 1.5931 |
| G06 | UNG | -1.3143 | -15.5817 | -9.5958 | 1.7392 |
| G07 | XPA | -1.0848 | 1.1002 | -1.0163 | 1.2858 |
| G08 | XPC | -1.1216 | -1.6011 | -1.1691 | 1.6471 |
| G09 | XRCC1 | -1.0294 | -2.4766 | -2.0942 | 1.5697 |
| G10 | XRCC2 | -1.0937 | -20.2261 | -11.6951 | 1.4195 |
| G11 | XRCC3 | 1.3647 | 1.2003 | -1.6742 | 1.0121 |
| G12 | ZAK | -1.192 | 8.0371 | 3.2422 | 1.0086 |
| H01 | B2M | -1.0137 | 8.818 | 10.7476 | 1.2756 |
| H02 | HPRT1 | 1.3835 | -3.4396 | -1.2931 | 1.5037 |
| H03 | RPL13A | 1.2623 | -1.3935 | -1.1418 | 1.8185 |
| H04 | GAPDH | -1.0465 | 1.3717 | -1.2661 | 1.9484 |
| H05 | ACTB | 1 | 1 | 1 | 1 |
| H06 | HGDC | -1.265 | -1.4694 | -1.1943 | -2.9983 |
| H07 | RTC | -1.2028 | -1.1355 | -1.168 | 1.6411 |
| H08 | RTC | -1.162 | -1.0761 | -1.1454 | 1.907 |
| H09 | RTC | -1.0973 | -1.0331 | -1.0914 | 1.9335 |
| H10 | PPC | -1.1898 | -1.161 | -1.2584 | 1.1571 |
| H11 | PPC | -1.106 | 1.0552 | -1.189 | 1.1911 |
| H12 | PPC | -1.1445 | -1.0656 | -1.1252 | 1.5966 |
