# Supplementary material for: DNA Damage Responses in Human Induced Pluripotent Stem Cells and Embryonic Stem Cells
Source: PLoS One. 2010 Oct 15;5(10):e13410. doi: 10.1371/journal.pone.0013410 (PMC2955528; doi:10.1371/journal.pone.0013410)
Supplement: Table S2 — p-values for fold difference in gene expression in designated cell line relative to human embryonic stem cells. (0.10 MB DOC) [file pone.0013410.s006.doc]

Table S1.

A: *p*-values (relative to ES cell expression): DNA damage signaling and cell cycle arrest genes.

| **Gene** | **IMR-90 iPS** | **AE iPS** | **IMR-90** | **TF** |
| --- | --- | --- | --- | --- |
| **ATM** | 0.57631 | 0.506295 | 0.085474 | 0.298853 |
| **ATR** | 0.224651 | 0.136748 | 0.00012 | 0.007757 |
| **BRCA1** | 0.696705 | 0.80286 | 0.039688 | 0.173745 |
| **CCNH** | 0.94397 | 0.428333 | 0.224058 | 0.853943 |
| **CDK7** | 0.955632 | 0.259967 | 0.819881 | 0.682147 |
| **CHEK1** | 0.987316 | 0.118751 | 0.031091 | 0.103799 |
| **CHEK2** | 0.859196 | 0.844377 | 0.054215 | 0.106662 |
| **GADD45A** | 0.328035 | 0.92211 | 0.183808 | 0.045834 |
| **GTSE1** | 0.648524 | 0.406655 | 0.069732 | 0.255467 |
| **HUS1** | 0.960966 | 0.949327 | 0.059013 | 0.309369 |
| **MNAT1** | 0.955009 | 0.478443 | 0.255932 | 0.611328 |
| **PCNA** | 0.850991 | 0.233539 | 0.023314 | 0.121541 |
| **RAD1** | 0.771902 | 0.536311 | 0.291469 | 0.20101 |
| **RAD17** | 0.955772 | 0.473923 | 0.120408 | 0.406977 |
| **RAD9A** | 0.597626 | 0.537845 | 0.023091 | 0.122588 |
| **RBBP8** | 0.824502 | 0.345977 | 0.084385 | 0.28663 |
| **SMC1A** | 0.737524 | 0.272268 | 0.049401 | 0.094141 |
| **TP53** | 0.773965 | 0.377934 | 0.023513 | 0.098894 |

Red font emphasizes statistically significant *p-*values (*p*<0.05). Abbreviations: IMR-90 iPS – iPS line derived from IMR-90 fibroblasts. AE iPS – iPS line derived from amniotic epithelial cells. IMR-90 – a fibroblast cell line. TF – teratoma fibroblasts derived from human embryonic stem cell line WA07.

**B: *p***-value (relative to ES cell expression): DSB repair genes.

| **Gene** | **IMR-90 iPS** | **AE iPS** | **IMR-90** | **TF** |
| --- | --- | --- | --- | --- |
| **ABL1** | 0.949231 | 0.549173 | 0.504224 | 0.452685 |
| **ATM** | 0.57631 | 0.506295 | 0.085474 | 0.298853 |
| **ATR** | 0.224651 | 0.136748 | 0.00012 | 0.007757 |
| **BRCA1** | 0.696705 | 0.80286 | 0.039688 | 0.173745 |
| **FANCG** | 0.947707 | 0.562551 | 0.005944 | 0.042056 |
| **FEN1** | 0.398639 | 0.310912 | 0.000734 | 0.015698 |
| **XRCC6 (Ku70)** | 0.943276 | 0.654172 | 0.033858 | 0.123937 |
| **XRCC6BP1** | 0.754221 | 0.505813 | 0.069761 | 0.389662 |
| **LIG1** | 0.930717 | 0.546939 | 0.011995 | 0.067929 |
| **MRE11A** | 0.413568 | 0.515739 | 0.019145 | 0.046147 |
| **NBN (Nbs1)** | 0.973539 | 0.711284 | 0.053436 | 0.543504 |
| **PRKDC (DNA-PKcs)** | 0.821906 | 0.694589 | 0.00513 | 0.029441 |
| **RAD1** | 0.771902 | 0.536311 | 0.291469 | 0.20101 |
| **RAD17** | 0.955772 | 0.473923 | 0.120408 | 0.406977 |
| **RAD18** | 0.995641 | 0.545698 | 0.04567 | 0.203224 |
| **RAD21** | 0.916088 | 0.790471 | 0.071021 | 0.223934 |
| **RAD50** | 0.824122 | 0.711063 | 0.185977 | 0.696658 |
| **RAD51L1** | 0.982636 | 0.809205 | 0.084185 | 0.331463 |
| **RAD9A** | 0.597626 | 0.537845 | 0.023091 | 0.122588 |
| **RPA1** | 0.735362 | 0.536414 | 0.023445 | 0.072859 |
| **XRCC2** | 0.573091 | 0.769153 | 0.027158 | 0.066824 |
| **XRCC3** | 0.325782 | 0.994872 | 0.834946 | 0.68676 |

Red font emphasizes statistically significant *p-*values (*p*<0.05). Abbreviations: IMR-90 iPS – iPS line derived from IMR-90 fibroblasts. AE iPS – iPS line derived from amniotic epithelial cells. IMR-90 – a fibroblast cell line. TF – teratoma fibroblasts derived from human embryonic stem cell line WA07.

**C: *p*-values (relative to ES cell expression): MMR genes.**

| **Gene** | **IMR-90 iPS** | **AE iPS** | **IMR-90** | **TF** |
| --- | --- | --- | --- | --- |
| **ABL1** | 0.949231 | 0.549173 | 0.504224 | 0.452685 |
| **ANKRD17** | 0.934429 | 0.731614 | 0.09786 | 0.204471 |
| **EXO1** | 0.492576 | 0.522098 | 0.031245 | 0.095152 |
| **MLH1** | 0.416364 | 0.465582 | 0.081946 | 0.23796 |
| **MLH3** | 0.997759 | 0.965141 | 0.174521 | 0.328437 |
| **MSH2** | 0.857991 | 0.435612 | 0.023702 | 0.060512 |
| **MSH3** | 0.869539 | 0.269519 | 0.041186 | 0.430153 |
| **MUTYH** | 0.451588 | 0.259017 | 0.016515 | 0.056259 |
| **N4BP2** | 0.864914 | 0.672229 | 0.009036 | 0.031524 |
| **PMS1** | 0.937467 | 0.754491 | 0.081887 | 0.186824 |
| **PMS2** | 0.292682 | 0.415731 | 0.641265 | 0.838477 |
| **PMS2L3** | 0.725087 | 0.863888 | 0.818104 | 0.401013 |
| **TREX1** | 0.916034 | 0.528133 | 0.191454 | 0.035572 |

Red font emphasizes statistically significant *p-*values (*p*<0.05). Abbreviations: IMR-90 iPS – iPS line derived from IMR-90 fibroblasts. AE iPS – iPS line derived from amniotic epithelial cells. IMR-90 – a fibroblast cell line. TF – teratoma fibroblasts derived from human embryonic stem cell line WA07.

D: *p*-value (relative to ES cell expression): BER genes.

| **Gene** | **IMR-90 iPS** | **AE iPS** | **IMR-90** | **TF** |
| --- | --- | --- | --- | --- |
| **APEX1** | 0.881701 | 0.216286 | 0.085594 | 0.381403 |
| **MBD4** | 0.877766 | 0.361172 | 0.267382 | 0.230659 |
| **MPG** | 0.487583 | 0.456759 | 0.011145 | 0.068083 |
| **MUTYH** | 0.451588 | 0.259017 | 0.016515 | 0.056259 |
| **NTHL1** | 0.815198 | 0.135108 | 0.063159 | 0.177981 |
| **OGG1** | 0.816398 | 0.570708 | 0.116337 | 0.49135 |
| **UNG** | 0.540893 | 0.21115 | 0.033375 | 0.078898 |
| **XRCC1** | 0.811104 | 0.168127 | 0.029069 | 0.152095 |

Red font emphasizes statistically significant *p-*values (*p*<0.05). Abbreviations: IMR-90 iPS – iPS line derived from IMR-90 fibroblasts. AE iPS – iPS line derived from amniotic epithelial cells. IMR-90 – a fibroblast cell line. TF – teratoma fibroblasts derived from human embryonic stem cell line WA07.

E: *p*-value (relative to ES cell expression): NER genes.

| **Gene** | **IMR-90 iPS** | **AE iPS** | **IMR-90** | **TF** |
| --- | --- | --- | --- | --- |
| **ERCC1** | 0.467596 | 0.353567 | 0.240213 | 0.29478 |
| **ERCC2** | 0.3228 | 0.588618 | 0.124794 | 0.314321 |
| **RPA1** | 0.735362 | 0.536414 | 0.023445 | 0.072859 |
| **XPA** | 0.949872 | 0.933206 | 0.673024 | 0.879367 |
| **XPC** | 0.808056 | 0.690012 | 0.256061 | 0.601866 |
| **XRCC1** | 0.811104 | 0.168127 | 0.029069 | 0.152095 |

Red font emphasizes statistically significant *p-*values (*p*<0.05). Abbreviations: IMR-90 iPS – iPS line derived from IMR-90 fibroblasts. AE iPS – iPS line derived from amniotic epithelial cells. IMR-90 – a fibroblast cell line. TF – teratoma fibroblasts derived from human embryonic stem cell line WA07.
